# Expansion of the Inguinal Adipose Tissue Depot Correlates With Systemic Insulin Resistance in C57BL/6J Mice

**DOI:** 10.3389/fcell.2022.942374

**Published:** 2022-09-07

**Authors:** Claes Fryklund, Mathis Neuhaus, Björn Morén, Andrea Borreguero-Muñoz, Richard Lundmark, Karin G. Stenkula

**Affiliations:** ^1^ Department of Experimental Medical Science, Lund University, Lund, Sweden; ^2^ Integrative Medical Biology, Umeå University, Umeå, Sweden

**Keywords:** adipocytes, cell size, obesity, glucose transport, insulin, cytoskeleton

## Abstract

To accommodate surplus energy, the adipose tissue expands by increasing adipocyte size (hypertrophy) and number (hyperplasia). The presence of hypertrophic adipocytes is a key characteristic of adipose tissue dysfunction. High-fat diet (HFD) fed C57BL/6J mice are a commonly used model to study obesity and obesity-related complications. In the present study, we have characterized adipose plasticity, at both the cellular and tissue level, by examining the temporal development of systemic insulin resistance and adiposity in response to HFD-feeding for 4, 8, and 12 weeks (4w, 8w, and 12w). Within the same time frame, we examined systemic metabolic flexibility and adipose plasticity when switching from HFD- to chow-diet during the last 2 weeks of diet intervention (referred to as the reverse (REV) group: 4wREV (2w HFD+2w chow), 8wREV (6w HFD+2w chow), 12wREV (10w HFD+2w chow)). In response to HFD-feeding over time, the 12w group had impaired systemic insulin sensitivity compared to both the 4w and 8w groups, accompanied by an increase in hypertrophic inguinal adipocytes and liver triglycerides. After reversing from HFD- to chow-feeding, most parameters were completely restored to chow control levels for 4wREV and 8wREV groups. In contrast, the 12wREV group had a significantly increased number of hypertrophic adipocytes, liver triglycerides accumulation, and impaired systemic insulin sensitivity compared to chow-fed mice. Further, image analysis at the single-cell level revealed a cell-size dependent organization of actin filaments for all feeding conditions. Indeed, the impaired adipocyte size plasticity in the 12wREV group was accompanied by increased actin filamentation and reduced insulin-stimulated glucose uptake compared with chow-fed mice. In summary, these results demonstrate that the C57BL/6J HFD-feeding model has a large capacity to restore adipocyte cell size and systemic insulin sensitivity, and that a metabolic tipping point occurs between 8 and 12w of HFD-feeding where this plasticity deteriorates. We believe these findings provide substantial understanding of C57BL/6J mice as an obesity model, and that an increased pool of hypertrophic ING adipocytes could contribute to aggravated insulin resistance.

## 1 Introduction

Adipose tissue constitutes the main storage site of excess energy in the body and plays an important role in meeting the energy demand of other tissues. Diet-induced obesity mouse models, such as high-fat diet (HFD) feeding in C57BL/6J mice, are a well-established approach to study obesity and obesity-related complications, including systemic and peripheral insulin resistance (Surwit et al., 1998; Winzell and Ahren, 2004; Hansson et al., 2018). Several studies have shown that glucose tolerance in mice is impaired already after a few days of HFD-feeding and further deteriorates over time (Winzell and Ahren, 2004; Williams et al., 2014; Fisher-Wellman et al., 2016; Hansson et al., 2018). The immediate onset of systemic insulin resistance in response to HFD-feeding is linked to impaired hepatic insulin action, which is followed by peripheral insulin resistance in adipose and muscle tissue (Turner et al., 2013). The length of intervention, mouse strain used, and fat content of HFD vary greatly between studies (de Moura e Dias et al., 2021). Nevertheless, an increase in fat mass commonly correlates with impaired systemic glucose tolerance. Both fat mass and insulin resistance are adjustable, and are restored once mice are switched from HFD- to chow-feeding (Hansson et al., 2019). This also applies for long-term HFD-feeding if the period of chow-feeding is prolonged (Parekh et al., 1998). Interestingly, recovery from systemic glucose intolerance occurs before body weight and adiposity are fully reverted, suggesting that excess adipose tissue mass *per se* cannot account for HFD-induced systemic glucose intolerance (Shi et al., 2009; Kowalski et al., 2016). Still, obesity is one of the main risk factors for insulin resistance, type 2 diabetes, and cardiovascular disease, underscoring the importance of adipose tissue in maintaining normal glucose homeostasis.

Adipose tissue expands by increasing the total adipocyte number (hyperplasia) and adipocyte size (hypertrophy). The ability of adipose tissue to expand and reduce its mass in response to energy intake has been exemplified by mathematical modelling, illustrating a cell size-dependent growth/shrinkage rate of adipocytes (Jo et al., 2010). The impact of adipocyte size on cellular function and whole-body glucose homeostasis has been addressed in many studies, where large adipocytes are described as less insulin responsive (Franck et al., 2007; Laurencikiene et al., 2011; Acosta et al., 2016), and as contributing to impaired whole-body glucose homeostasis (Weyer et al., 2000). Indeed, increased adipocyte size has been shown to positively correlate with impaired systemic insulin sensitivity and impaired glucose tolerance in humans, independently of the degree of obesity (Acosta et al., 2016). Interestingly, both expansion and shrinkage of adipocyte size have been shown to correlate with changes in insulin signaling, intracellular actin organization and glucose transport (Hansson et al., 2019), emphasizing that not only cell size but also cellular function is restorable during reduced energy intake.

The aim of the present study was to characterize adipose plasticity in response to energy intake, at both cellular and tissue level, and further address its time dependency. Therefore, we examined the temporal development of systemic insulin resistance and adiposity in response to HFD-feeding (4, 8 and 12 weeks) in C57BL/6J mice. Within the same time frame, we assessed systemic metabolic flexibility (measured as insulin sensitivity and liver triglycerides) and adipose plasticity when switching from HFD- to chow-diet (the last 2 weeks). Additionally, cell-size distribution, actin organization and cellular insulin response were monitored in both subcutaneous (inguinal) and visceral (epididymal) adipocytes to examine depot-specific alterations.

## 2 Materials and Methods

### 2.1 Antibodies

Antibodies raised against insulin receptor substrate 1 (IRS-1) and acetyl-CoA carboxylase (ACC) were from Cell Signaling Technologies (Danvers, United States). Heat shock protein (HSP) 90 antibody was from BD Transduction Laboratories (Franklin Lakes, United States). Phosphorylated (S3) Cofilin-1 antibody was from Santa Cruz Biotechnology (Dallas, United States). GLUT4 antibody was kindly provided by Sam Cushman (NIH, United States). Phospho-specific antibodies raised against Akt substrate of 160 kDa (AS160) pT642, IRS-1 pY612, and Akt pS473 were from Cell Signaling Technologies (Danvers, United States).

### 2.2 Animals and Diet Intervention

**FIGURE 1:**
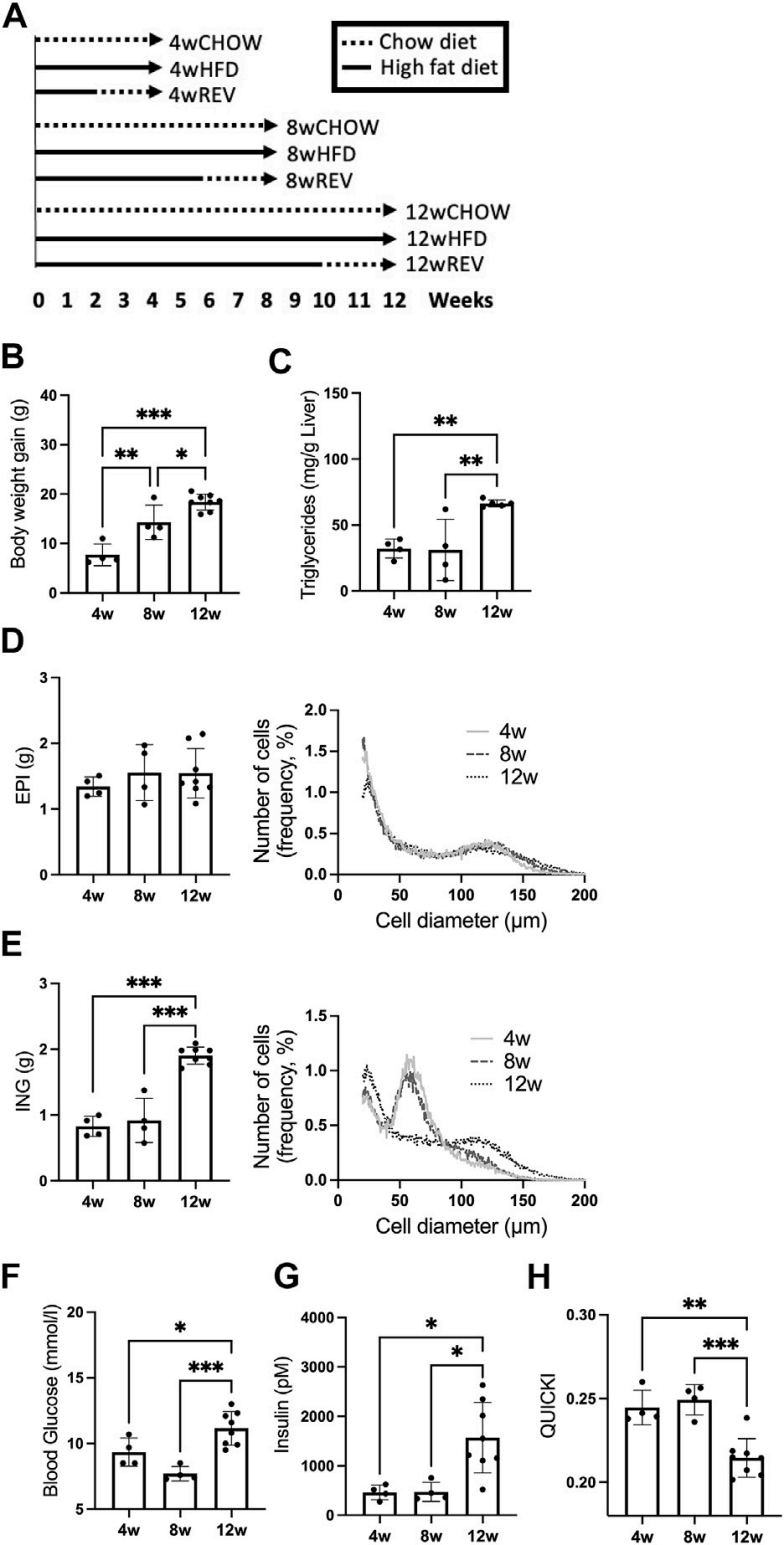
Study outline and time-resolved effect of HFD-feeding on systemic metabolism and adipocyte expansion. **(A)** Study outline for time-resolved analysis of HFD-feeding and reversal from HFD to chow. C57BL/6J mice, 9 weeks old, were subjected to time-resolved analysis for 4, 8, and 12 weeks of diet intervention, each time point including the following three groups: CHOW, chow-feeding throughout the diet intervention, *n* = 7 (4w), *n* = 10 (8w), *n* = 9 (12w). HFD, high-fat diet feeding throughout the diet intervention, *n* = 4 (4w), *n* = 4 (8w), *n* = 8 (12w). REV, high-fat diet feeding, switched to chow-feeding during the final 2 weeks of diet intervention, *n* = 11 (4w), *n* = 5 (8w), *n* = 10 (12w). **(B–H)** Mice subjected to HFD-feeding for 4, 8, or 12 weeks. Final **(B)** body-weight gain and **(C)** liver triglycerides (mg/g liver). Final fat depot weight and adipose cell-size distribution of **(D)** EPI and **(E)** ING. Cell-size distribution graphs display the percentage of cells (y-axis) and cell diameter (µm) (x-axis), 4w and 8w *n* = 4/time point, 12w *n* = 5. **(F)** Blood glucose (mM), **(G)** serum insulin (pM), and corresponding **(H)** QUICKI. Data are displayed as mean ± SD and one-way ANOVA, Tukey’s post-hoc test, was used as statistical analysis. Significance was determined according to *p ≤ 0.05, **p ≤ 0.01 and ***p ≤ 0.001.

Male C57BL/6J mice (Taconic, Ry, Denmark) were 9 weeks of age at the start of the diet-intervention. Mice were on a 12-hour light cycle with non-restricted food and water supply and were acclimatized 1 week before start. Mice (*n* = 4–11/group) were fed either chow (4, 8 or 12 weeks), high-fat diet (HFD) (#D12492, 60E% fat, Research Diets, New Brunswick, United States), or HFD (2, 6, or 10 weeks) followed by 2 weeks of chow [defined as reverse (REV)]. The study outline and feeding groups are shown in Figure 1A. Body weight of individual mice was measured weekly. Mice were fasted 2 h prior to termination, where after liver and adipose tissue (epididymal (EPI), inguinal (ING), and retroperitoneal (RETRO) adipose tissue) were excised, weighed, and used for adipose cell size distribution analyses, adipocyte isolation, or directly placed in liquid nitrogen for mRNA extraction. Blood samples were collected from the *vena saphena* to measure blood glucose and serum insulin levels. All animal procedures were approved by the Malmö/Lund Committee for Animal Experiment Ethics, Lund, Sweden.

### 2.3 Serum Analysis

Fasting (2 h) blood glucose levels were measured (OnetouchUltra2 (Lifescan, Milpitas, CA, United States), and fasting (2 h) insulin levels were assayed in terminal serum samples using ELISA (Mercodia, Uppsala, Sweden). Quantitative insulin sensitivity check index (QUICKI) was calculated as described previously: QUICKI = 1/[log(fasting insulin (μU/ml))+log(fasting glucose (mg/dl))], using the conversion factor 1 μU/mL = 6.00 p.m. for insulin (Katz et al., 2000).

### 2.4 Liver Triglyceride Content

Liver triglyceride concentration was determined according to Abcam protocol (Ab65336). In short, ∼100 mg frozen liver sample was homogenized using a Dounce homogenizer in buffer containing 5% (w/v) NP-40. The homogenized samples were heated at 90°C for 3 min, cooled down for 15 min at room temperature, reheated at 90°C for 3 min, and then centrifuged at 10000xg for 2 min at 20°C. The supernatant was collected and placed in a 96-well plate with triglyceride reagent (Thermo Scientific, #TR22421) for triglyceride concentration determination.

### 2.5 Isolation of Primary Adipocytes

Primary adipocytes were isolated from adipose tissue depots as described previously (Rodbell, 1964). The cells were suspended in Krebs Ringer Bicarbonate HEPES (KRBH) buffer, pH 7.4, containing 200 nM adenosine and 3% (w/v) Bovine serum albumin (BSA).

### 2.6 Glucose Uptake

Glucose uptake was determined as previously described (Gliemann et al., 1984). Cells (7.5 % (v/v) suspension) were incubated with or without 10 nM insulin in KRBH buffer in triplicates for 30 min, followed by the addition of D-^14^C(U)-glucose (2.5 μL/ml, NEC042, Perkin Elmer, Waltham, United States), and an additional 30 min of incubation. The uptake was terminated by centrifugation of 300 µL of each cell suspension in microtubes containing 80 µL dinonylphtalate oil. The cell fraction was collected, dissolved in scintillation fluid (Ultima Gold, Perkin Elmer), and subjected to scintillation counting.

### 2.7 Western Blot Analysis

For western blot analysis of primary adipocytes, cells were washed twice with KRBH medium without BSA and subsequently lysed in lysis buffer containing 50 mM Tris/HCl pH 7.5, 1 mM EGTA, 1 mM EDTA, 0.27 M sucrose, 1% NP-40, and complete protease- and phosphatase inhibitor cocktail (Roche, Basel, Switzerland). Lysates were centrifuged for 10 min at 13000xg, and protein concentrations were determined using the Bradford method. Samples were subjected to polyacrylamide gel electrophoresis and electro-transfer to nitrocellulose membranes. Membranes were blocked with non-fat dry milk [5% (w/v)] and probed (overnight, 4°C) with the indicated antibodies. Detection was performed using horseradish peroxidase-conjugated secondary antibodies and enhanced chemiluminescence reagent. The signal was visualized using a BioRad Image camera (Biorad, Hercules, United States). All data were normalized using HSP90 as a loading control.

### 2.8 Adipose Cell-Size Distribution

At termination, adipose tissue samples (8 × 4 mg/sample) were obtained from the inguinal and epididymal depots and fixed with osmium for adipose cell-size distribution analysis using a Multisizer 4e Coulter Counter (Beckman-Coulter, Brea, United States) as described previously (McLaughlin et al., 2007). For each sample, the size of 6,000 particles was counted and run in technical duplicates. Data were analyzed using linear bins (400 bins, bin-size 0.55 µm) Multisizer3 version 3.53. Large adipocytes in Figure 4C and Supplementary Figure S1 were defined as cells larger than cell diameter corresponding to the 95th percentile from 4wCHOW [>95 (EPI) and >70 (ING) µm diameter]. The total number of adipocytes per fat depot was calculated from the results obtained from the Coulter counter. The average volume (ml) of 6,000 cells and the fat depot weight (g) was converted to total cells per fat depot by using the density *ρ* = 0.915 g/ml as a conversion factor for adipose tissue mass. The total number of large adipocytes was calculated by multiplying the total number of adipocytes by the fraction of cells larger than >95 (EPI) and >70 (ING) µm diameter.

### 2.9 RT-qPCR Analysis

Adipose tissue samples were obtained from the inguinal and epididymal adipose tissue depots (*n* = 3–5). Total RNA was extracted from frozen adipose tissue using a guanidinium thiocyanate-phenol-chloroform extraction method (miRNeasy mini kit (Qiagen #74104)). RNA integrity was assessed using NanoDrop. RT-qPCR was performed using the Quantifast SYBR Green RT-PCR kit (Qiagen #204156), and Quantitect primer assays for *18S* (QT02448075), CD44 antigen (*Cd44*; QT00173404), collagen type VI alpha 3 (*Col6a3*; QT00251671) and CD68 (*Cd68*; QT00254051). Primer sequences are considered proprietary information by Qiagen. mRNA expression levels were measured using a StepOnePlus real-time thermal cycler (Applied Biosystems Waltham, United States) and quantitated using the ΔΔCT method as described by Livak and Schmittgen (Livak and Schmittgen, 2001). 18S rRNA was used for normalization throughout. Statistical analysis was carried out on ΔCT values.

### 2.10 Total Internal Reflection Fluorescence Microscopy

For total internal reflection fluorescence (TIRF) imaging we used a commercial TIRF system based on a Nikon Ti-E eclipse microscope equipped with a 100× Apo TIRF DIC oil immersion objective NA of 1.49 (Nikon Instruments Inc.), an iXon Ultra DU-897 EMCCD camera (Andor Technology Ltd.), and four main laser lines, 405 (Cube, Coherent Inc), 488 (Melles-Griot), 561 (Sapphire, Coherent Inc), and 640 (Cube, Coherent Inc) with corresponding filter sets. Isolated cells were fixed using 4% PFA and labelled with phalloidin using a buffer containing 0.05% saponin for 1 h.

For quantification of the grade of actin polymerization, an ImageJ plugin ridge detection was used to trace actin filaments, detected with phalloidin stain, in TIRF microscopy images. Standard values were used, and the threshold adjusted until most of the visible actin was traced. Images were exported using the “make binary” command. A region of interest (ROI) of roughly ¼ of the cell was chosen and used to obtain consistent data on the grade of polymerization. This ROI was used on all cells to obtain the area of binary cell traces within the threshold.

### 2.11 Statistical Analysis

Statistical analyses were performed by one-way ANOVA, Tukey’s post-hoc test, or Student’s t-test, using GraphPad Prism (GraphPad Software Inc.) software. Significance was determined according to *p ≤ 0.05, **p ≤ 0.01, ***p ≤ 0.001. All data are displayed as mean ± SD. Linear correlation analysis was performed by calculating Pearson’s correlation coefficient (r).

## 3 Results

### 3.1 Time-dependent Progression of Systemic Insulin Resistance and Adipose Tissue Expansion During High-Fat Diet-Feeding

To characterize the time-dependent influence of HFD-feeding on insulin sensitivity and adiposity, we examined systemic parameters and adipose tissue expansion using three different feeding periods: 4, 8 and 12 weeks (4w, 8w and 12w) of HFD. We found body-weight gain to be the only parameter that increased significantly between 4w and 8w (Figure 1B). Liver triglycerides were significantly increased between 8w and 12w (Figure 1C). The epididymal (EPI) fat weight was similar at all time points, while the inguinal (ING) fat expanded and doubled in weight comparing 8w with 12w group (Figures 1D,E). Using the Coulter counter, we assessed EPI and ING tissue expansion at a cellular level. The EPI cell-size distribution did not differ over time with HFD-feeding and showed an accumulation of large (>95 µm diameter) adipocytes already after 4w (Figure 1D, right panel). The ING depot displayed primarily medium-sized adipocytes (50–70 µm diameter) after 4w and 8w, then accumulated a substantial fraction of large (>70 µm diameter) adipocytes after 12w (Figure 1E, right panel). Taking the total adipose tissue depot weight into account, the number of large, hypertrophic adipocytes increased significantly in the ING, but not EPI depot after 12w (Supplementary Figure S1). Further, both fasting blood glucose and serum insulin, and thus insulin sensitivity (determined as QUICKI) were impaired in the 12w group compared to 4w and 8w groups (Figures 1F–H). Thus, in our cohort of mice, we observed a markedly impaired systemic insulin sensitivity between 8w to 12w of HFD-feeding which coincided with an accumulation of hypertrophic ING adipocytes and increased ING fat mass.

### 3.2 Time-dependent Changes in Systemic Insulin Sensitivity and Adipose Plasticity When Switching From High-Fat Diet- to Chow-Feeding

Next, we wanted to address the capacity of systemic insulin sensitivity and adiposity to recuperate after HFD-feeding. Therefore, groups of mice were switched from HFD to chow-feeding for the last 2 weeks of intervention, referred to as reverse (REV): 4wREV (2w HFD+2w chow), 8wREV (6w HFD+2w chow), 12wREV (10w HFD+2w chow). Mice fed chow only (CHOW) for 4/8/12 weeks were included as a control (see study outline in Figure 1A).

**FIGURE 2:**
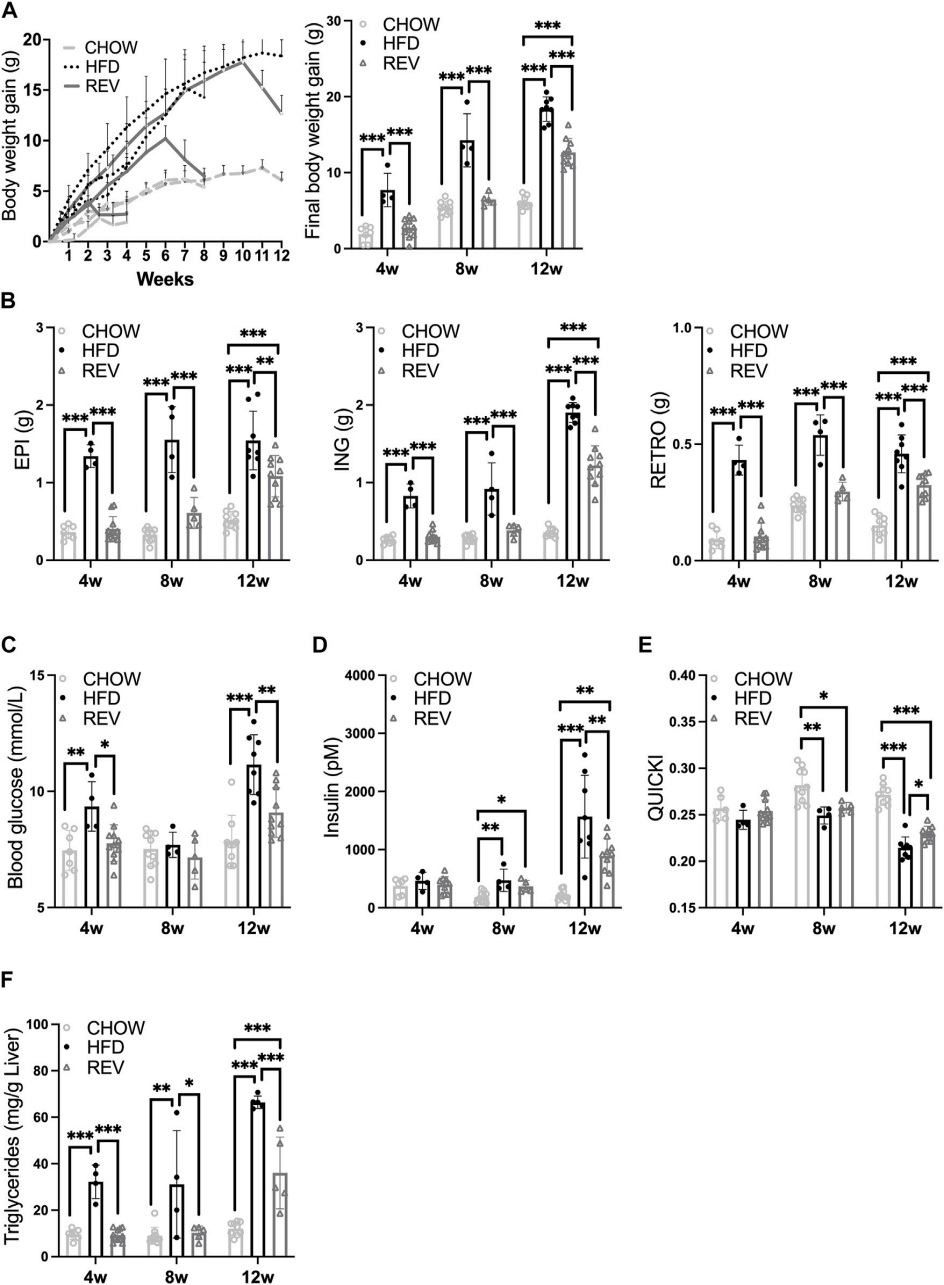
Time-resolved analysis of systemic metabolic response and adipose tissue plasticity after switching from HFD to chow. Mice were subjected to diet intervention for 4, 8 or 12 weeks, each timepoint containing the following three groups: CHOW, HFD and REV (see Figure 1 for study outline). Note, the same data from HFD are displayed in Figure 1. **(A)** Body-weight gain was monitored weekly (left panel) and at termination (right panel). **(B)** Final fat depot weight (EPI, ING, and RETRO). **(C)** Blood glucose (mM), **(D)** serum insulin (pM) and corresponding **(E)** QUICKI. **(F)** Liver triglycerides (mg/g liver). Data are displayed as mean±SD and one-way ANOVA, Tukey’s post-hoc test, was used as statistical analysis of differences between CHOW, HFD and REV for each time point (4, 8, and 12w). Significance was determined according to *p ≤ 0.05, **p ≤ 0.01 and ***p ≤ 0.001.

**FIGURE 3:**
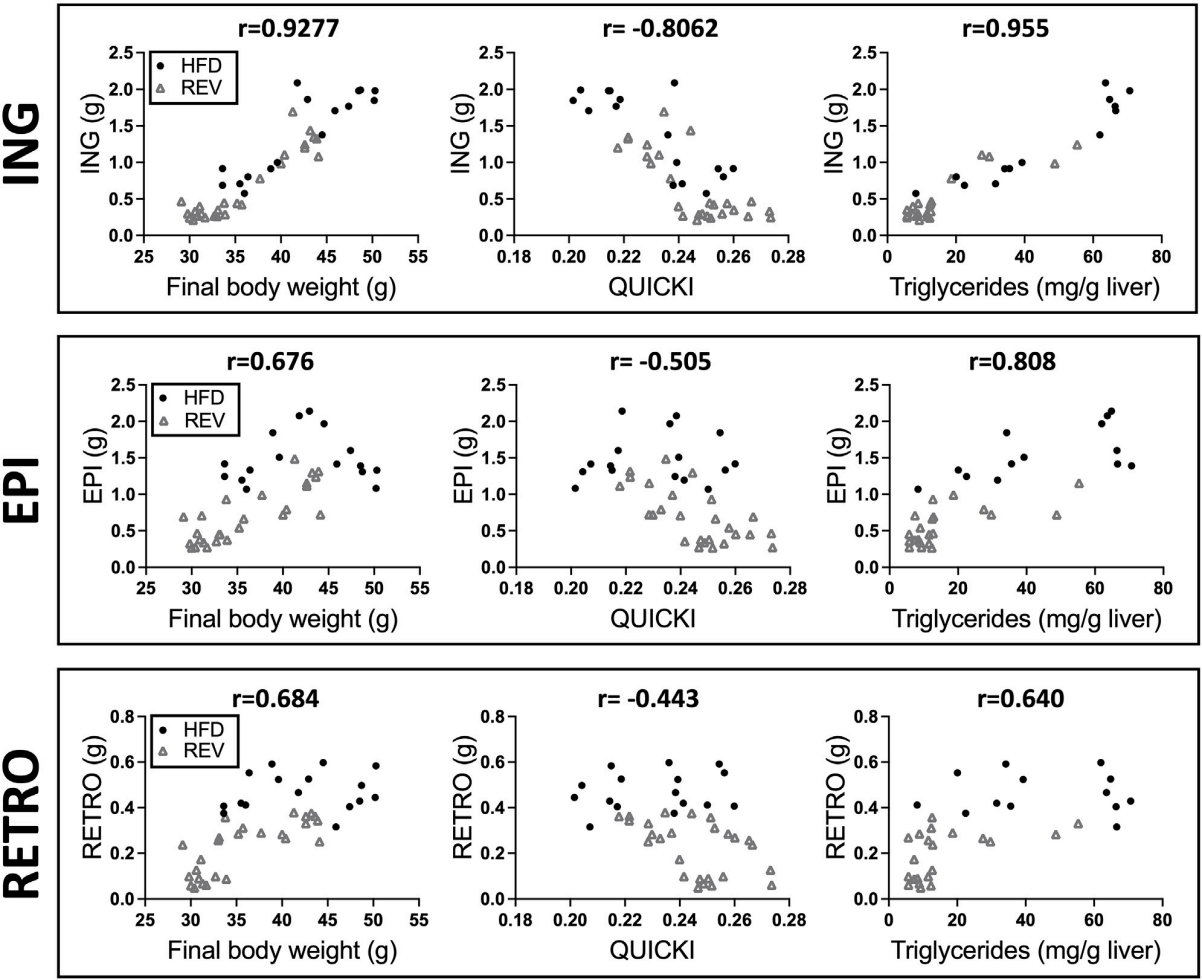
Correlation analysis between different fat depots and body weight, QUICKI or liver triglycerides. Scatter plot displaying correlation between fat depot mass (g) and final body weight, QUICKI or liver triglycerides, (ING, top panel), (EPI, middle panel), and (RETRO, bottom panel). Pearson correlation with corresponding r value is displayed in each graph. The data are obtained from HFD (black dot) and REV (gray square) groups from 4, 8 and 12w of intervention (see study outline Figure 1).

Body-weight gain and fat depot weights were fully reverted to CHOW levels for 4wREV and 8wREV, while the 12wREV had significantly increased body-weight gain and fat depot weights compared to CHOW (Figures 2A,B). Fasting blood glucose levels were not significantly different at any time point comparing REV to CHOW, while serum insulin was increased for both 8wREV and 12wREV compared to CHOW (Figures 2C,D). The 8wREV and 12wREV also displayed impaired insulin sensitivity, measured as QUICKI, compared to CHOW (Figure 2E). Still, insulin sensitivity was significantly lower for both the 12wHFD and 12wREV in comparison to 8wHFD, indicating that insulin sensitivity progressively worsened during this feeding period (from 8 to 10 weeks of HFD) and that reversing the diet at this time point only partially improved insulin sensitivity (QUICKI: 0.215 for 12wHFD, 0.23 for 12wREV and 0.25 for 8wHFD) (Figure 2E). Liver triglycerides were reverted to CHOW for 4wREV and 8wREV but not for the 12wREV group, which had levels comparable to the 4wHFD and 8wHFD (Figure 2F). Interestingly, ING adipose tissue mass correlated with body weight, liver triglycerides and QUICKI to a higher degree than both EPI and retroperitoneal (RETRO) adipose tissue mass (Figure 3).

Changes in adipose tissue mass involve remodeling of the extracellular matrix (Sun et al., 2013) and an increased infiltration of immune cells, which is considered to contribute to systemic insulin resistance (Kodama et al., 2012). Therefore, we examined the mRNA expression of collagen, type VI, alpha 3 (*Col6a3)*, encoding one of the most abundant collagens in the adipose tissue matrix, as well as cell-surface glycoprotein CD44, known to induce immune cell infiltration and to correlate with insulin resistance (Kodama et al., 2012), and also *CD68*, a macrophage marker. In the EPI adipose tissue depot, we found increased mRNA levels of *Col6a3, CD44* and *CD68* in the 12wHFD group compared to CHOW, which stayed elevated also in the 12wREV group (Supplementary Figure S2A). In contrast, no changes in these targets were observed in the ING adipose tissue depot (Supplementary Figure S2A). Notably, in chow-fed mice, the expression levels of *Col6a3, CD44* and *CD68* were relatively higher in ING compared with EPI (Supplementary Figure S2A). We also examined the temporal (4w, 8w and 12w) expression of *CD44* in the RETRO fat depot. There was an increase in *CD44* expression in response to HFD, which was reverted to chow level for the 4w and 8w REV group, but not for the 12w REV group (Supplementary Figure S2B).

Together, while the metabolic flexibility in the 8wREV group was similar to the 4wREV group, a much more blunted recovery of adiposity and insulin sensitivity was observed in the 12wREV group. The data also suggest that the mass of the ING depot is more strongly correlated with systemic insulin sensitivity than the EPI and RETRO depots during prolonged HFD-feeding.

### 3.3 Cell-Size Distribution and Cellular Actin Density When Switching From High-Fat Diet- to Chow-Feeding

**FIGURE 4:**
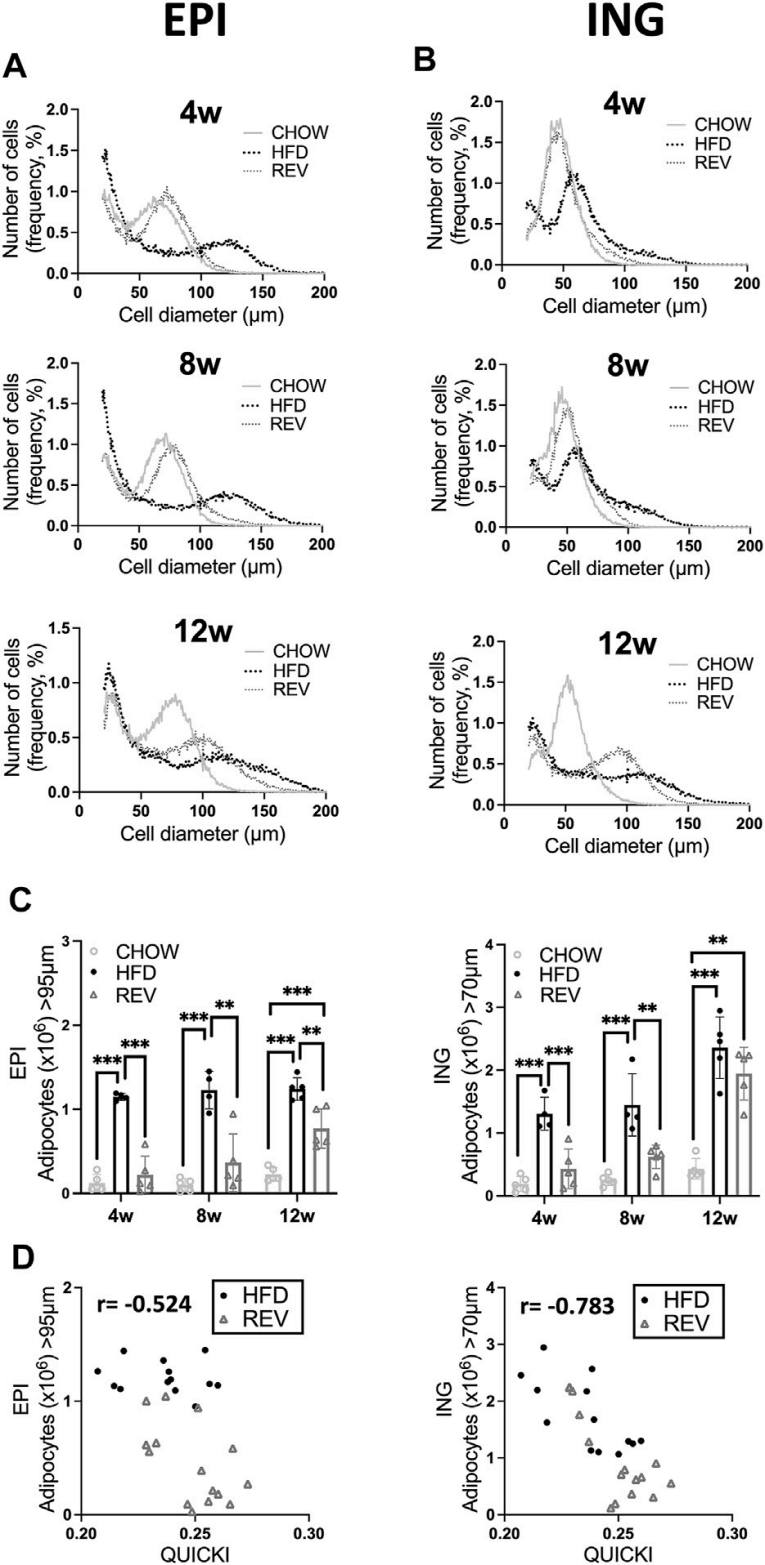
Time resolved analysis of cell-size distribution from epididymal and inguinal adipose tissue. Mice were subjected to diet intervention for 4, 8 or 12 weeks, each timepoint containing the following three groups: CHOW, HFD and REV (see Figure 1 for study outline). Adipocyte cell-size distribution of **(A)** EPI and **(B)** ING fat depot, obtained from Coulter counter analysis, displaying CHOW, HFD and REV groups from 4 weeks (top panel), 8 weeks (middle panel) and 12 weeks (bottom panel). Cell-size distribution graphs display the percentage of cells (y-axis) and cell diameter (µm) (x-axis), n = 4–5/condition and time point. **(C)** Total number of hypertrophic adipocytes (EPI > 95 μm, ING > 70 μm, definition and calculation see method Section 2.8) in EPI (left) and ING (right) adipose tissue. **(D)** Scatter plot displaying correlation between number of hypertrophic adipocytes (defined as above) and insulin sensitivity (QUICKI) in EPI (left panel) and ING (right panel) fat depot, with corresponding r value from Pearson correlation. Data are displayed as mean ± SD. One-way ANOVA, Tukey’s post-hoc test, was used as statistical analysis of differences between CHOW, HFD and REV for each time point (4, 8, and 12w). Significance was determined according to *p ≤ 0.05, **p ≤ 0.01 and ***p ≤ 0.001.

To examine how changes in fat depot mass affected adipocyte size, we assessed cell-size distribution in the EPI and ING fat depots. The accumulation of large, hypertrophic adipocytes [>95 (EPI) and >70 (ING) µm diameter] observed after 4w and 8w of HFD-feeding was reduced to similar levels as observed in CHOW in the REV groups for both fat depots (Figures 4A–C). In contrast, the 12wREV group had a cell-size distribution resembling that observed with 12wHFD rather than CHOW (Figures 4A,B bottom panel). Quantification of the total number of hypertrophic adipocytes at the tissue level in the EPI depot (Figure 4C) revealed a significant decrease in the number of hypertrophic adipocytes when reversing the diet, independent of the duration of HFD-feeding. However, the ING depot had similar number of hypertrophic adipocytes even after reversing the diet at the 12 weeks’ time point (Figure 4C). Further, the number of hypertrophic ING adipocytes displayed a strong correlation with systemic insulin sensitivity (measured as QUICKI), which was not observed in hypertrophic EPI adipocytes (Figure 4D). These results suggest that the hypertrophic inguinal adipocytes that develop after prolonged HFD-feeding might negatively influence systemic metabolism.

**FIGURE 5:**
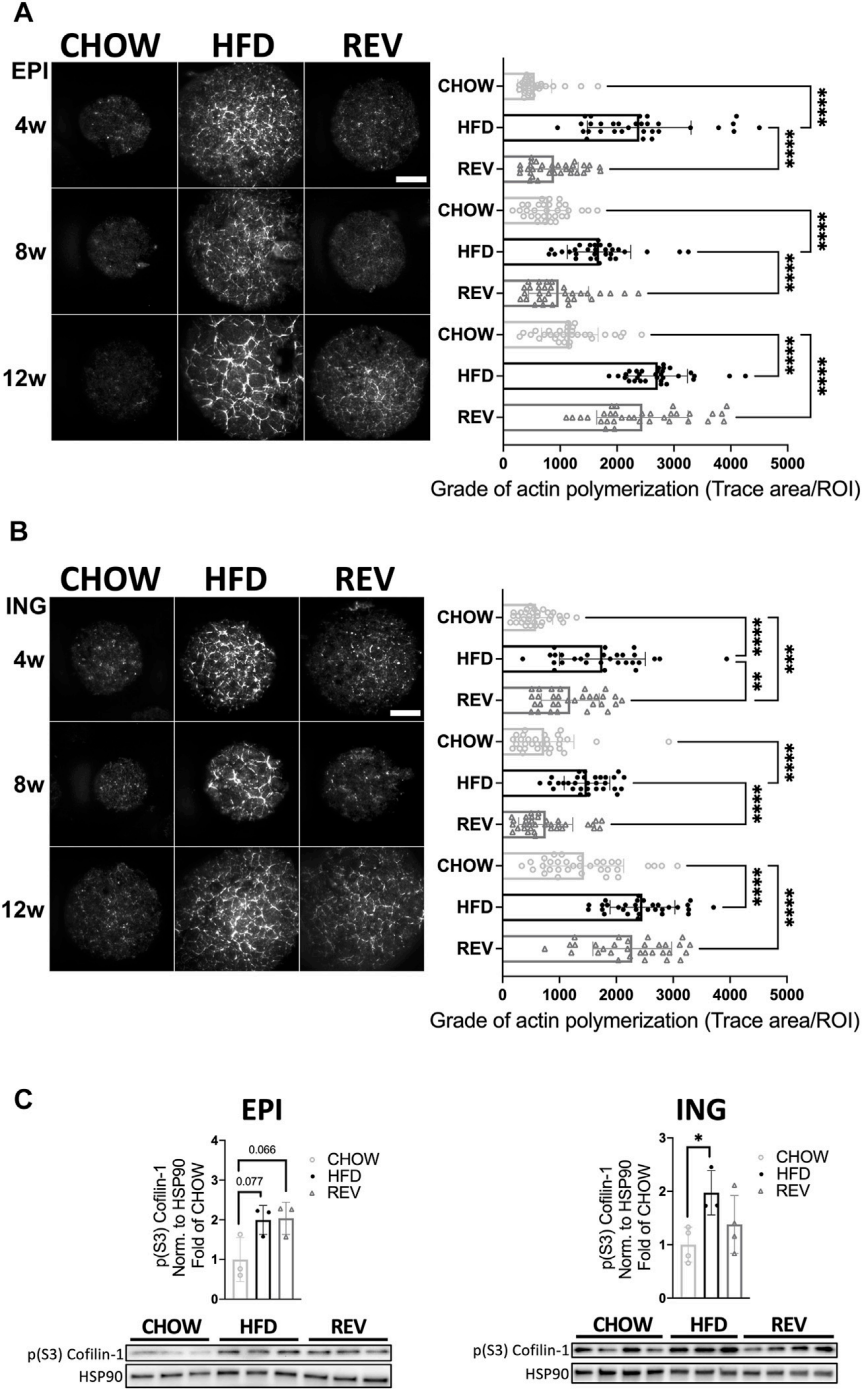
Time resolved analysis of actin polymerization in primary adipocytes following diet intervention. TIRF microscopy of primary adipocytes isolated from EPI **(A)** and ING **(B)** fat depot stained with phalloidin to detect filamentous actin. Representative images (left panel) from CHOW, HFD and REV during 4w, 8w, or 12w of diet intervention, and corresponding quantification (right panel) for each time point, *n* = 29–35 cells/condition. **(C)** Western blot analysis and corresponding quantification of phosphorylated (S3) Cofilin-1 in lysates from EPI (left panel) and ING (right panel) adipocytes after 12w of diet. Values are expressed as fold of CHOW and HSP90 was used as a loading control. Data are displayed as mean±SD. One-way ANOVA, Tukey’s post-hoc test, was used as statistical analysis of differences between CHOW, HFD and REV for each time point (4, 8, and 12w). Significance was determined according to *p ≤ 0.05, **p ≤ 0.01 and ***p ≤ 0.001.

Further, we have previously shown that adipose cell size correlates positively with the density of filamentous actin and that both cell size and density of filamentous actin are reversible when switching from HFD- to chow-feeding during short feeding intervention (corresponding to the 4 weeks feeding used herein) (Hansson et al., 2019). To investigate if adipose plasticity was also sustained after prolonged HFD-feeding, we used TIRF microscopy and phalloidin labelling to detect filamentous actin in single adipocytes isolated from CHOW, HFD, and REV groups. Indeed, the density of polymerized actin was significantly increased in HFD compared to CHOW at all time points tested, in both EPI and ING adipocytes (Figures 5A,B). At the 4w and 8w time points, the level of filamentous actin was significantly increased in HFD compared to REV, while they displayed similar levels comparing 12wREV to 12wHFD. By western blot analysis, we found a trend (*p* = 0.07) towards increased levels (∼2-fold) of phosphorylated Cofilin-1 (pS3), known to regulate the severing of polymeric actin, in the EPI 12wHFD and 12wREV groups compared with CHOW, which supports the increased actin density observed by microscopy (Figure 5C).

Together, these data illustrate that the plasticity in adipocyte size mirrors adaptations in adipose tissue mass, and that actin is structured in a cell-size dependent fashion independent of the feeding regime.

### 3.4 Cellular Insulin Response After Diet Reversal From High-Fat Diet- to Chow-Feeding

**FIGURE 6:**
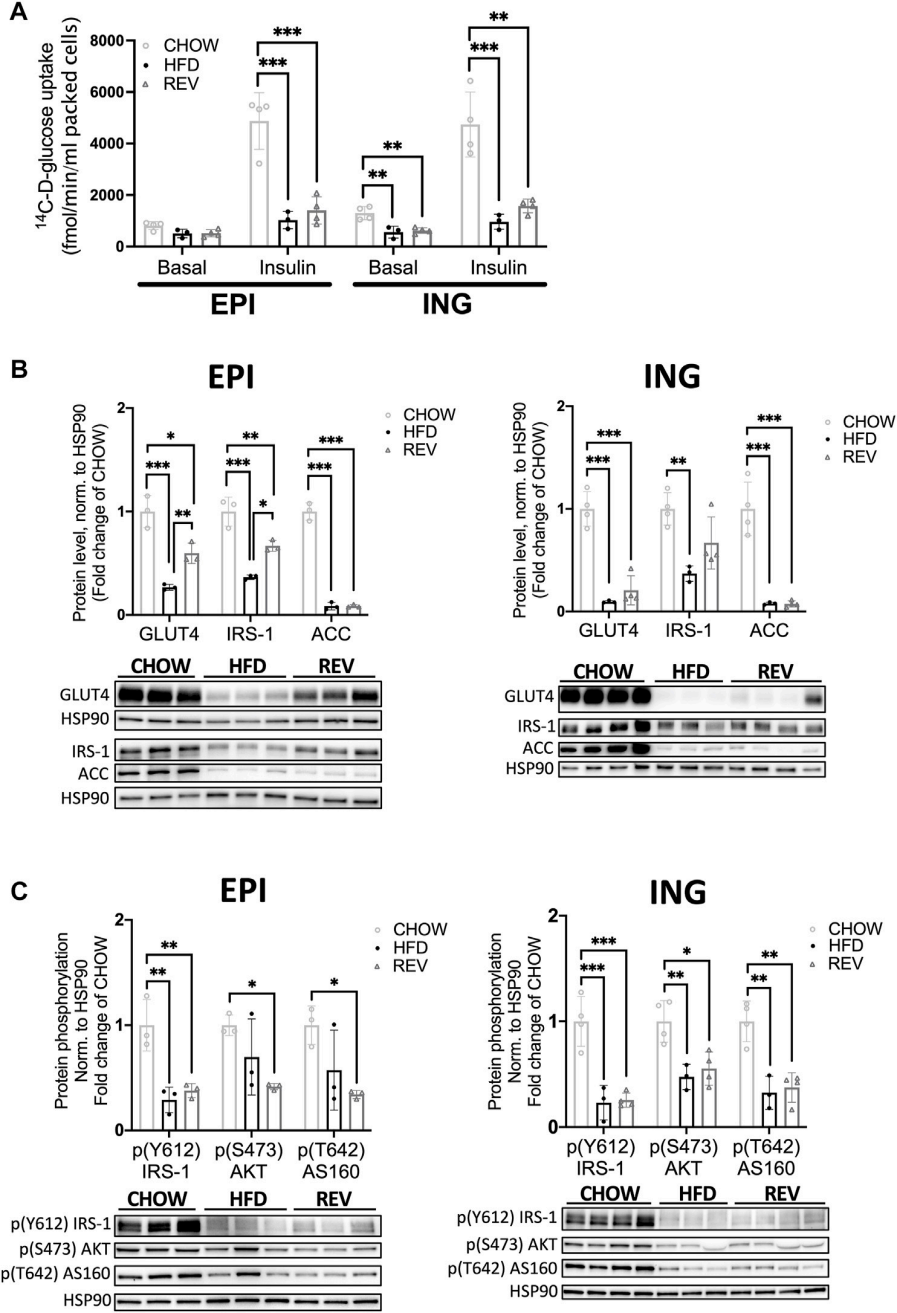
Insulin-stimulated glucose uptake in primary adipocytes following 12 weeks of diet intervention. **(A)** Non (basal) and insulin stimulated glucose uptake in primary adipocytes isolated from EPI or ING fat depot following 12-week diet-intervention of CHOW, HFD and REV. In short, adipocytes were isolated and then non-stimulated (basal) or stimulated with insulin (10 nM) for 30 min before adding a glucose-tracer for additional 30 min incubation. Glucose uptake is expressed as femtomole glucose uptake per min per ml packed cells. *n* = 3–4/condition. **(B)** Western blot analysis from 12-week diet-intervention and corresponding quantification of GLUT4, IRS-1 and ACC from isolated EPI (left panel) and ING (right panel) adipocytes. **(C)** Western blot analysis from 12-week diet-intervention and corresponding quantification of phosphorylation of IRS-1 (Y612), AKT (S473) and AS160 (T642) from non-stimulated (basal) EPI (left panel) and ING (right panel) adipocytes. Values are expressed as fold of CHOW and HSP90 was used as a loading control. Data are displayed as mean±SD and one-way ANOVA, Tukey’s post-hoc test, was used as statistical analysis. Significance was determined according to *p ≤ 0.05, **p ≤ 0.01 and ***p ≤ 0.001.

Next, we set out to examine whether the impaired systemic insulin sensitivity after 12w of HFD and the blunted recovery in the 12wREV group were also seen at the cellular level, by using a glucose tracer assay in isolated adipocytes. Both EPI and ING adipocytes from HFD-fed mice displayed a sharp drop (∼80%) in insulin-stimulated glucose uptake compared to CHOW (Figure 6A). Adipocytes obtained from the REV group had significantly lower insulin-stimulated uptake compared to CHOW, even though ING adipocytes showed a trend towards improved insulin sensitivity comparing HFD to REV (*p* = 0.089) (Figure 6A). Notably, ING adipocytes from both HFD and REV had significantly lowered non-stimulated (Basal) uptake compared with CHOW (Figure 6A).

Further, the protein levels of insulin receptor substrate 1 (IRS-1), the intermediate downstream target of the insulin receptor, and the insulin-regulated glucose transporter (GLUT)4 protein were clearly decreased in response to HFD in both EPI and ING adipocytes (Figure 6B). Both IRS-1 and GLUT4 were modestly but significantly increased in the REV group in EPI but not ING adipocytes (Figure 6B). Further, the expression of Acetyl Co-A carboxylase (ACC), a protein that catalyzes *de novo* lipid synthesis, was suppressed by ∼90% with HFD. ACC levels remained low also in the REV group in both fat depots (Figure 6B). Further, the temporal expression of IRS-1, GLUT4 and ACC in EPI adipocytes revealed that 12wREV, but not 4wREV or 8wREV, had significant reduced protein levels compared to CHOW (Supplementary Figure 5). Thus, the poor cellular recovery of insulin responsiveness after reversing the diet in the 12 week group could reflect the presence of a high fraction of hypertrophic adipocytes (shown in Figures 4A,B). We also examined the insulin signaling pathway and found that the phosphorylation of IRS-1 (Y612), AKT (S473) and AS160 (T642) were significantly reduced in the 12w REV group compared to chow in non-stimulated (basal) EPI and ING adipocytes (Figure 6C), consistent with the reduced basal glucose uptake that we observed.

## 4 Discussion

In the current study, we report several novel observations that illustrate the capacity of C57BL/6J mice to adjust adiposity and insulin sensitivity in response to diet, and how this capacity changes over time. After 12 weeks of HFD-feeding, we observed a significant increase in liver triglyceride levels, reduced insulin sensitivity, a substantial expansion of the ING fat depot and an accumulation of hypertrophic ING adipocytes. The negative correlation between ING fat mass and insulin sensitivity was somewhat unexpected as the ability to expand subcutaneous fat in humans has been suggested to be protective against metabolic diseases (Tchernof and Despres, 2013). Fat depot transplantation and fat removal studies in mice also support that the ING fat depot is protective against metabolic diseases while the opposite is true for the EPI fat depot (Hocking et al., 2008; Foster et al., 2013; Franczyk et al., 2021). Most transplantation studies are conducted using ING fat that, based on length of HFD-feeding, does not exhibit very large ING hypertrophic adipocytes at the time of transplant. Therefore, there is a possibility that contrary results would be found if these studies were to be repeated using ING fat that contained very large hypertrophic adipocytes. Further, the increased lipid deposits in liver and ING depot reported herein may be related to a restricted capacity of the EPI depot to expand further. Such an impairment has previously been linked to adipose tissue inflammation, adipocyte apoptosis, hepatic steatosis and deteriorating systemic insulin sensitivity (Strissel et al., 2007). We found an increased mRNA expression of *CD68* and *CD44* in the EPI, but not ING fat depot, following 12 weeks of HFD-feeding, which supports that inflammation primarily occurs in the EPI depot. Notably, both *CD44* and *CD68* maintained upregulated also in the REV group suggesting that inflammation is not reversible at this timepoint. Our finding that the EPI depot reached a maximal expansion at body weights around 40–45 g and thereafter declined despite further increase in body weight, is consistent with findings in previous studies (see Supplementary Figure S3) (Strissel et al., 2007; van Beek et al., 2015). The decrease in EPI fat mass is likely related to increased apoptosis (Strissel et al., 2007; Jo et al., 2010; van Beek et al., 2015). In contrast, the ING fat depot and cell sizes expanded significantly after prolonged HFD-feeding. The large population of hypertrophic adipocytes could account for the negative correlation between ING fat mass and insulin sensitivity shown in Figure 4. Thus, the role ascribed to the ING depot as a metabolic sink, protecting against metabolic disease (Tchernof and Despres, 2013) may be diminished at this point.

We also examined the capacity for adiposity and insulin sensitivity to recuperate after reversing from HFD to chow diet. At the 12 weeks’ time point, mice had an impaired capacity to recuperate systemic metabolism and adiposity when switching from HFD- to chow-feeding, compared with the shorter times tested. Interestingly, the 8wREV group had smaller adipocyte sizes than observed in 4wHFD in both EPI and ING adipocytes (see Supplementary Figure S4, left panel). This was only found in EPI, not ING, adipocytes when comparing the 12wREV group with the 8wHFD group (see Supplementary Figure S4, right panel), which pinpoints fat depot-specific differences in the capacity to adjust to energy intake. Further, we have previously reported changes in actin organization mirror adipocyte size during shorter feeding interventions (Hansson et al., 2019). Herein, we could confirm that this is also true during more extended feeding periods. After 12 weeks of HFD-feeding we observed an impairment in insulin-stimulated glucose uptake in isolated adipocytes, which was not recovered by reversing the diet. This is most likely related to the abundance of large adipocytes (relatively to chow) and the increased density of actin filaments. Indeed, numerous studies have highlighted that actin turnover plays a vital role in promoting insulin-mediated GLUT4 exocytosis (Lopez et al., 2009; Yang et al., 2014). Possibly, decreased GLUT4 exocytosis is caused by a shift towards increased filamentous actin, required to maintain cellular integrity of the expanding adipocyte (Rivero et al., 1996). Interestingly, the partial restoration of IRS-1 and GLUT4 protein levels in EPI adipocytes after reversing the diet was not sufficient to improve insulin-stimulated glucose uptake. Thus, factors other than GLUT4 expression must contribute to the lowered insulin response, again emphasizing that actin dynamics could provide a mechanistic explanation for hypertrophic adipocyte dysfunction.

Numerous studies have addressed changes in adiposity and glucose homeostasis in response to diet-induced obesity in mice. However, there are several varying parameters, for example, mice strains, diet composition, length of feeding (de Moura e Dias et al., 2021). A study using an experimental design comparable to the 12 weeks’ group used herein, described a resistance to fat loss after reversing the diet, which was suggested to arise from leptin insufficiency (Shi et al., 2009). Despite having increased adiposity compared with chow, the reversal of HFD to chow-diet resulted in restored glucose tolerance (Shi et al., 2009). We found similar changes with respect to adiposity and reduced adipocyte sizes but observed significantly higher insulin levels. This discrepancy could be related to the difference in dietary fat content (45% versus 60% in our study).

The time of diet-intervention is an important factor for the outcome and herein we only evaluated the plasticity during a short reversing period. The outcome may have been different if we had prolonged the reversed feeding period, in line with a previous report where mice fed HFD for 4 months, followed by 4 months of low-fat, completely recovered (Parekh et al., 1998). Still, our study gives insight into the cell-size distribution and characteristics of subcutaneous and visceral adipose tissue, when metabolic flexibility and adipose tissue plasticity start to decline during HFD-feeding. In support of our protocol, it was mentioned in the study by Shi et al. (2009) that 2 weeks of diet reversal had been evaluated as a time point where body weight was confirmed to be stabilized. A limitation of our study design was the assessment of systemic insulin sensitivity. In order to correlate insulin sensitivity and adipose tissue parameters from the same individual, we used fasting insulin and glucose levels to calculate QUICKI as a measurement of insulin sensitivity, which serves as a valid measurement but does not fully dissect glucose tolerance (Bowe et al., 2014).

In summary, our results demonstrate that the C57BL/6J HFD-feeding model has a large capacity to restore adipocyte cell size and systemic insulin sensitivity, but also emphasize that a metabolic tipping point occurs between 8 and 12w of HFD-feeding, where these parameters are not restored at the same rate. Furthermore, at this time point we observed that the expansion of the ING depot, both in total mass and adipocyte size, coincides with deterioration of systemic insulin sensitivity. In conclusion, we believe these findings provide substantial understanding of C57BL/6J mice as an obesity model, and that an increased pool of hypertrophic ING adipocytes could contribute to aggravated insulin resistance.

## Data Availability

The raw data supporting the conclusions of this article will be made available by the authors, without undue reservation.
