# Supplementary material for: Expansion of the Inguinal Adipose Tissue Depot Correlates With Systemic Insulin Resistance in C57BL/6J Mice
Source: Front Cell Dev Biol. 2022 Sep 7;10:942374. doi: 10.3389/fcell.2022.942374 (PMC9489915; doi:10.3389/fcell.2022.942374)
Supplement: Supplementary file 1 [file Table1.DOCX]

**Supplementary Figure S1**

Number (x10^6^) of large adipocytes in EPI (left) and ING (right) fat depot following 4, 8, or 12 weeks of HFD-feeding. Large adipocytes were defined as cells having a cell diameter larger than the corresponding 95^th^ percentile from 4wCHOW (>95 (EPI) and >70 (ING) µm diameter). Number of large adipocytes was calculated from the results obtained from the Coulter counter: the average volume (ml) of 6000 cells and the fat depot weight (g) was converted to total cells per fat depot by using the density ρ=0.915g/ml as a conversion factor, multiplied by the fraction of cells larger than >95 (EPI) and >70 (ING) µm diameter. Data are displayed as mean±SD and one-way ANOVA, Tukey's post-hoc test, was used as statistical analysis. Significance was determined according to *=p≤0.05.

**Supplementary Figure S2**

**(A)** Relative mRNA levels of *CD44,* *Col6a3* and *CD68* in EPI and ING adipose tissue from the 12 week’s intervention group (12wCHOW, 12wHFD, 12wREV). **(B)** Relative mRNA levels of *CD44* in RETRO adipose tissue from the 4, 8 and 12 week’s intervention groups (4/8/12w CHOW, HFD, REV). mRNA levels were measured using two-step RT-qPCR, *18S* rRNA used as a reference, and relative gene expression to EPI CHOW was calculated using 2^–∆∆Ct^ method. Data are displayed as mean±SD and one-way ANOVA, Tukey's post-hoc test, was used as statistical analysis. Significance was determined according to *=p≤0.05, **=p≤0.01 and ***=p≤0.001.

**Supplementary Figure S3**

Scatter plot displaying individual mice following 4, 8 or 12 weeks of HFD-feeding. Left graph shows correlation between final body weight (BW) (x-axis) and EPI weight (y-axis). Right graph shows correlation between final body weight (BW) (x-axis) and systemic insulin sensitivity (QUICKI) (y-axis). The number next to each data point represent final body weight ranked from lowest (1) to highest (16) body weight.

**Supplementary Figure S4**

Comparison of cell-size distribution, fat depot weight, blood glucose, serum insulin, QUICKI and liver triglycerides in 4wHFD versus 8wREV (left panel), and 8wHFD versus 12wREV (right panel). Data are displayed as mean±SD and one-way ANOVA, Tukey's post-hoc test, was used as statistical analysis. Significance was determined according to *=p≤0.05, **=p≤0.01 and ***=p≤0.001.

**Supplementary Figure S5**

Western blot analysis and corresponding quantification displaying temporal (4w (top), 8w (middle), and 12w (bottom)) expression of GLUT4, IRS-1 and ACC in EPI adipocytes from CHOW, HFD and REV. Values are expressed as fold of CHOW and HSP90 was used as a loading control. Data are displayed as mean±SD and one-way ANOVA, Tukey's post-hoc test, was used as statistical analysis. Significance was determined according to *=p≤0.05, **=p≤0.01 and ***=p≤0.001.

**Supplementary Figure S6**

Uncropped image with merged ladder for the following western blot targets: HSP90, IRS-1, ACC, phosphorylated (S3) Cofilin-1 and GLUT4 from **(A)** EPI and **(B)** ING isolated adipocytes obtained from the 12 week’s feeding group (12wCHOW, 12wHFD, 12wREV). HSP90, p(Y612) IRS-1, p(S473) AKT and p(T642) AS160 from non-stimulated **(C)** EPI and **(D)** ING adipocytes the 12 week’s feeding group (12wCHOW, 12wHFD, 12wREV). HSP90, IRS-1, ACC, and GLUT4 from isolated EPI adipocytes obtained from the **(E)** 4 week’s and **(F)** 8 week’s feeding groups (CHOW, HFD, REV).
