# Supplementary material for: Expansion of the Inguinal Adipose Tissue Depot Correlates With Systemic Insulin Resistance in C57BL/6J Mice
Source: Front Cell Dev Biol. 2022 Sep 7;10:942374. doi: 10.3389/fcell.2022.942374 (PMC9489915; doi:10.3389/fcell.2022.942374)
Supplement: Supplementary file 2 [file Presentation1.PPTX]

## Slide 1
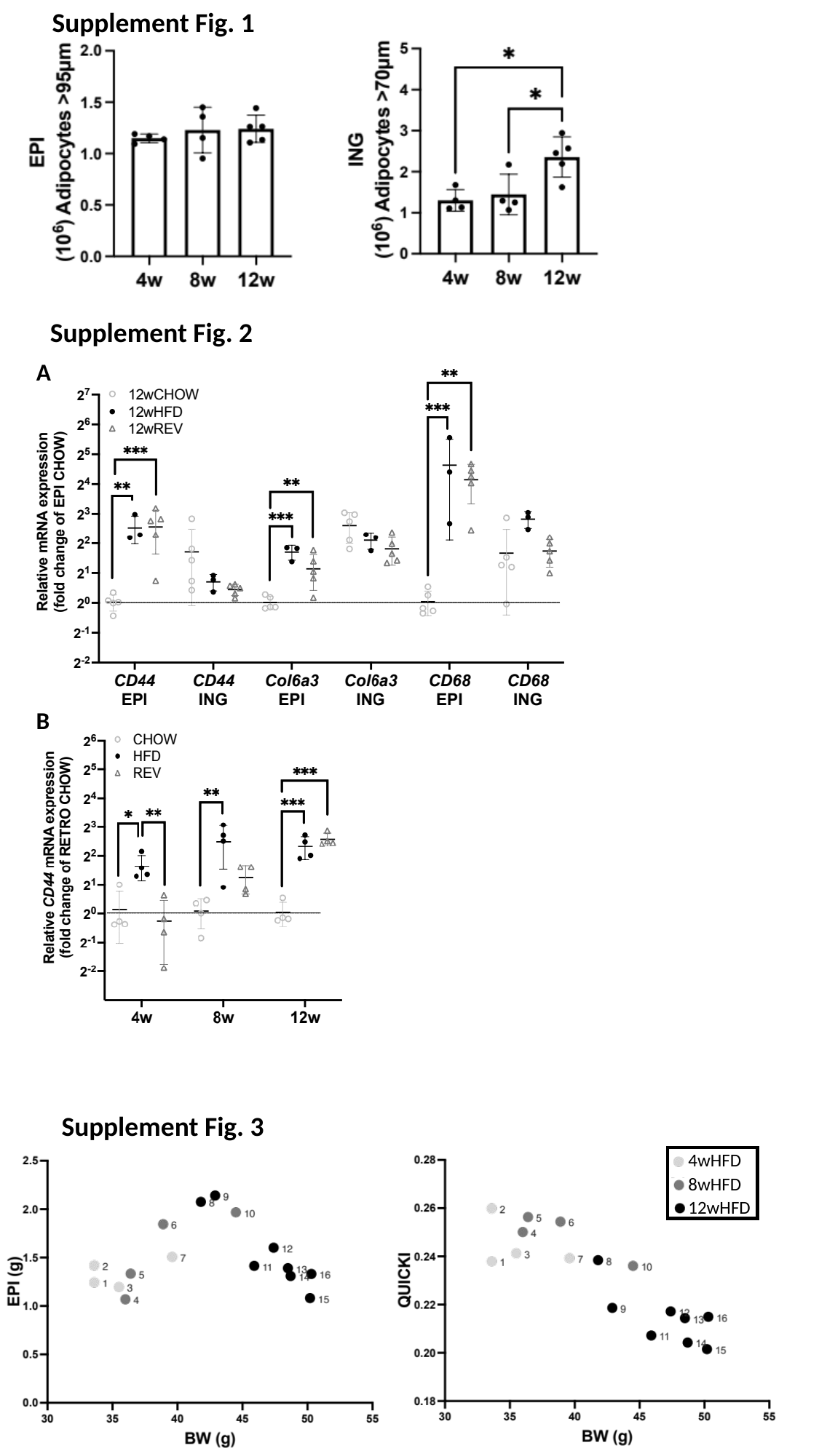

Supplement Fig. 1
Supplement Fig. 2
A
B
Supplement Fig. 3
4wHFD
8wHFD
12wHFD

## Slide 2
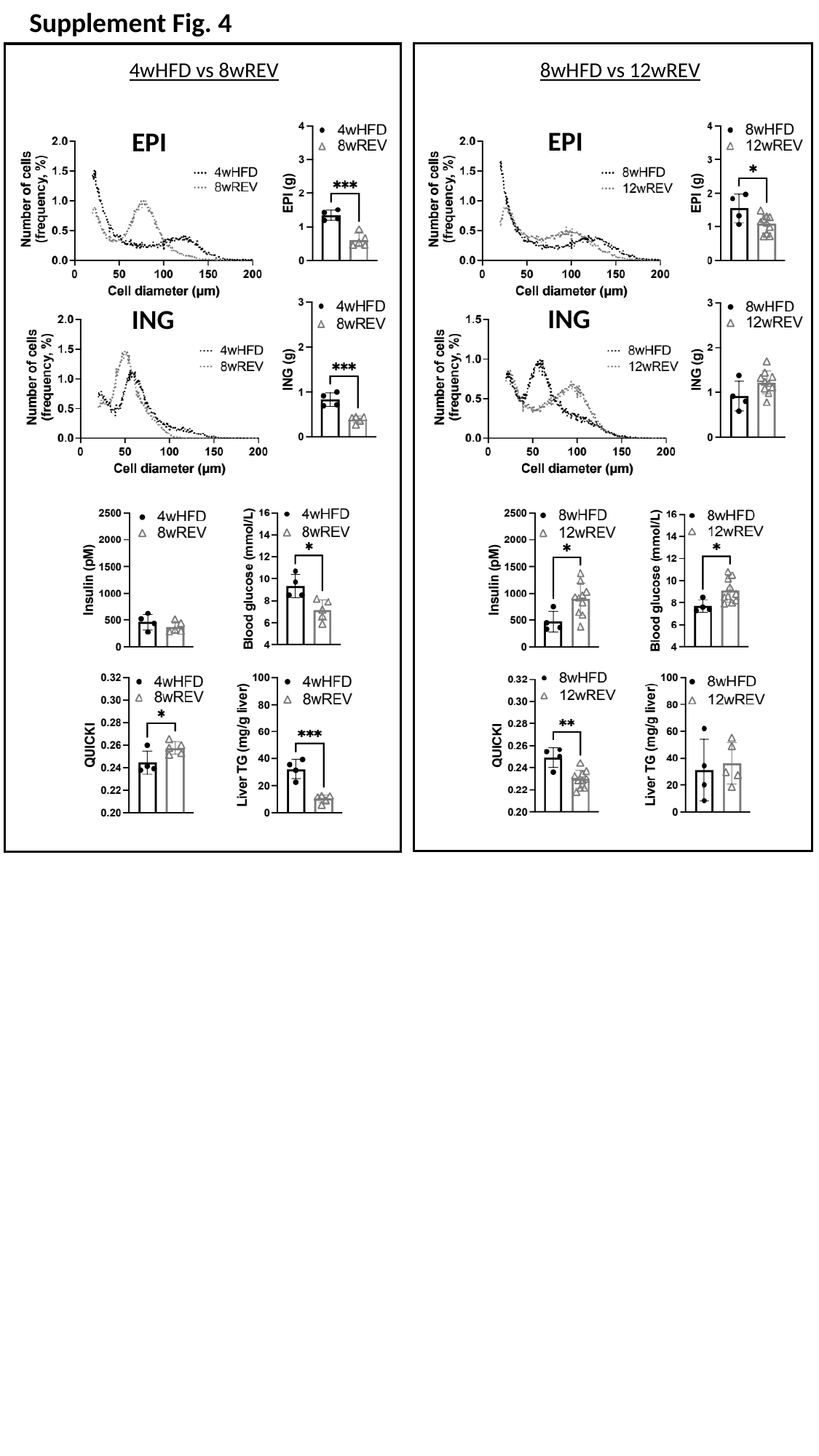

Supplement Fig. 4
4wHFD vs 8wREV
8wHFD vs 12wREV
EPI
EPI
ING
ING

## Slide 3
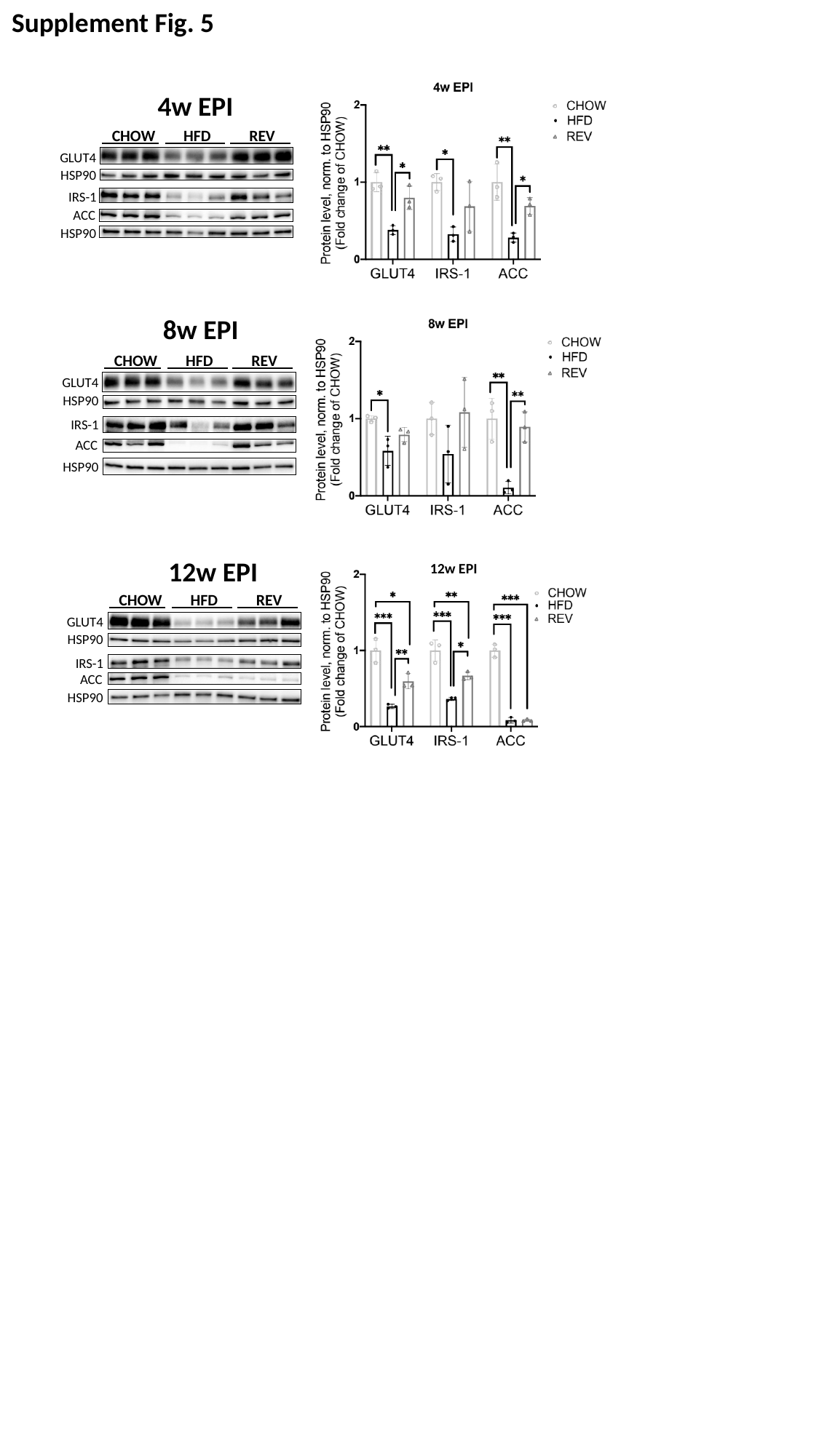

Supplement Fig. 5
4w EPI
CHOW
HFD
REV
GLUT4
HSP90
IRS-1
ACC
HSP90
8w EPI
CHOW
HFD
REV
GLUT4
HSP90
IRS-1
ACC
HSP90
12w EPI
CHOW
HFD
REV
GLUT4
HSP90
IRS-1
ACC
HSP90
12w EPI
