# Supplementary material for: Expansion of the Inguinal Adipose Tissue Depot Correlates With Systemic Insulin Resistance in C57BL/6J Mice
Source: Front Cell Dev Biol. 2022 Sep 7;10:942374. doi: 10.3389/fcell.2022.942374 (PMC9489915; doi:10.3389/fcell.2022.942374)

Supplement Fig. 6A

Western Blot uncropped, merged with ladder

EPI adipocytes 12w group: IRS-1, ACC, pCofilin-1 (S3), GLUT4

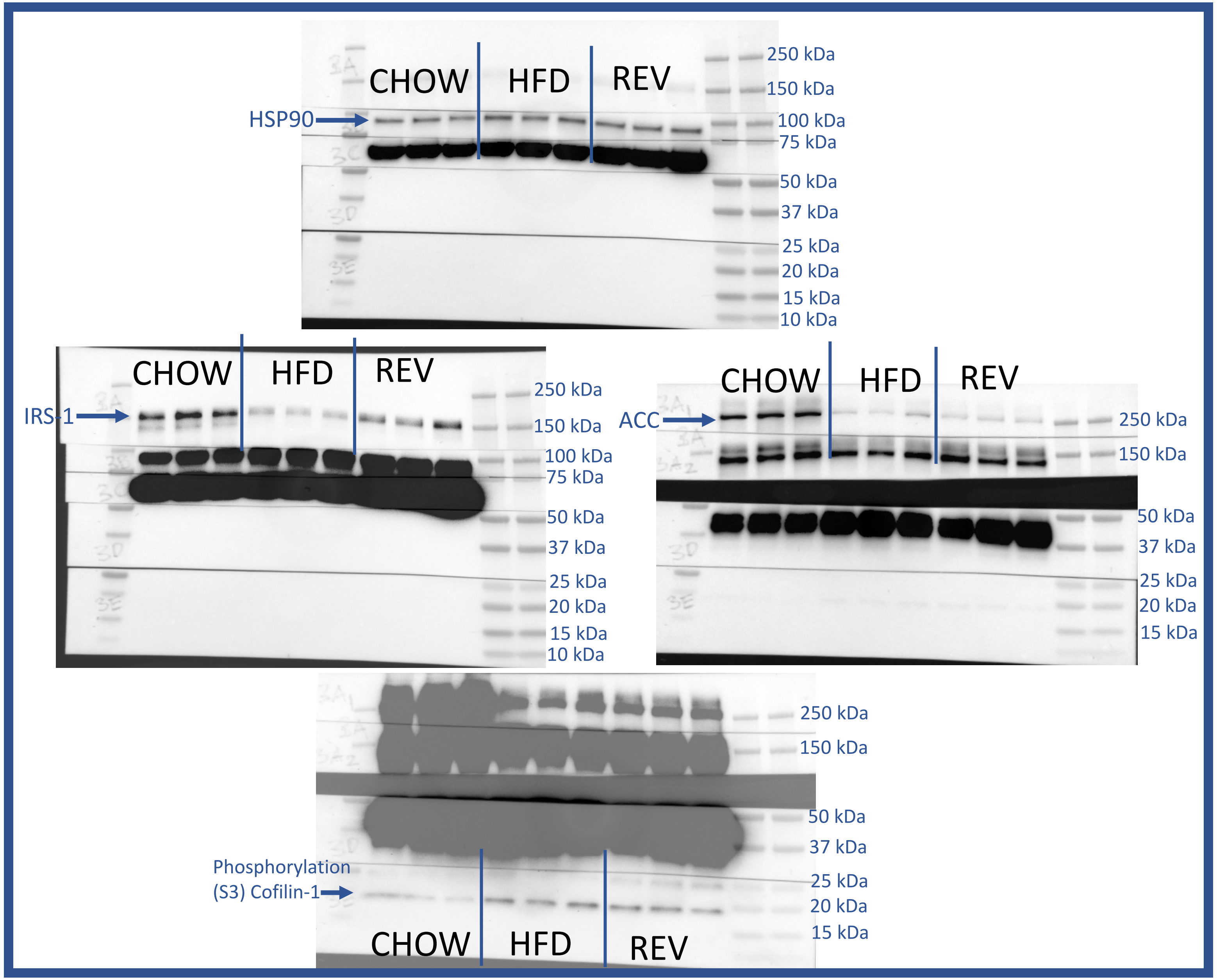

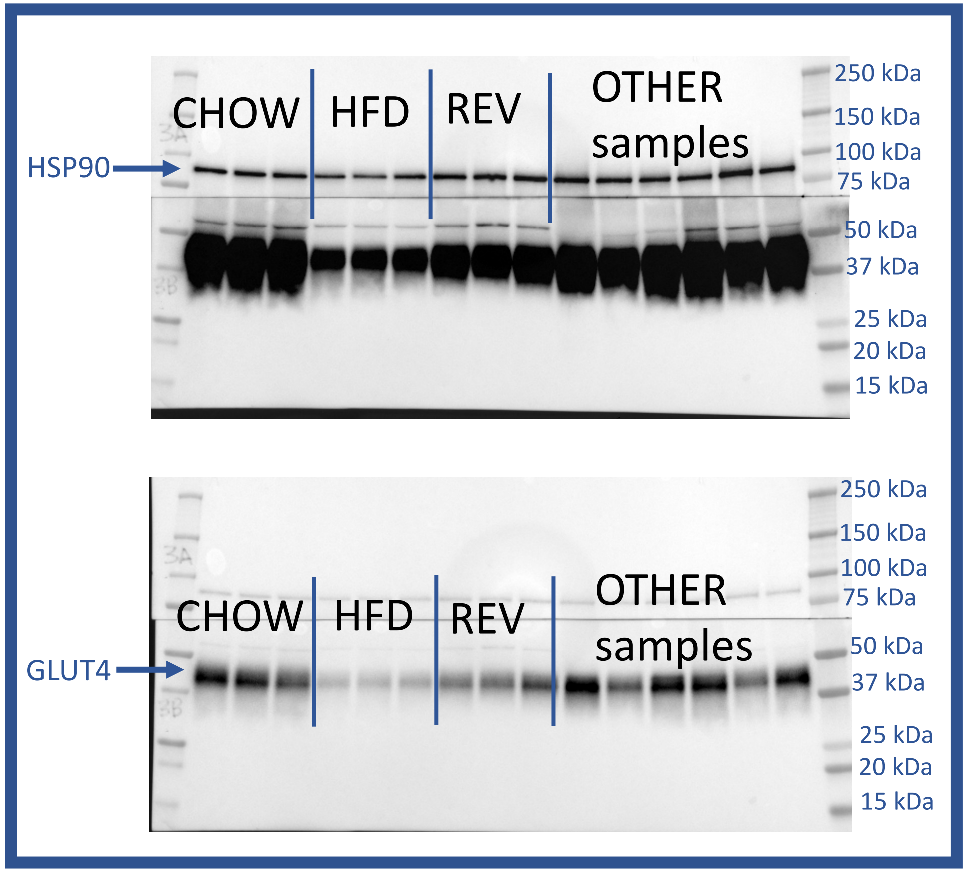

Supplement Fig. 6B

Western Blot uncropped, merged with ladder

ING adipocytes 12w group: IRS-1, ACC, pCofilin-1 (S3), GLUT4

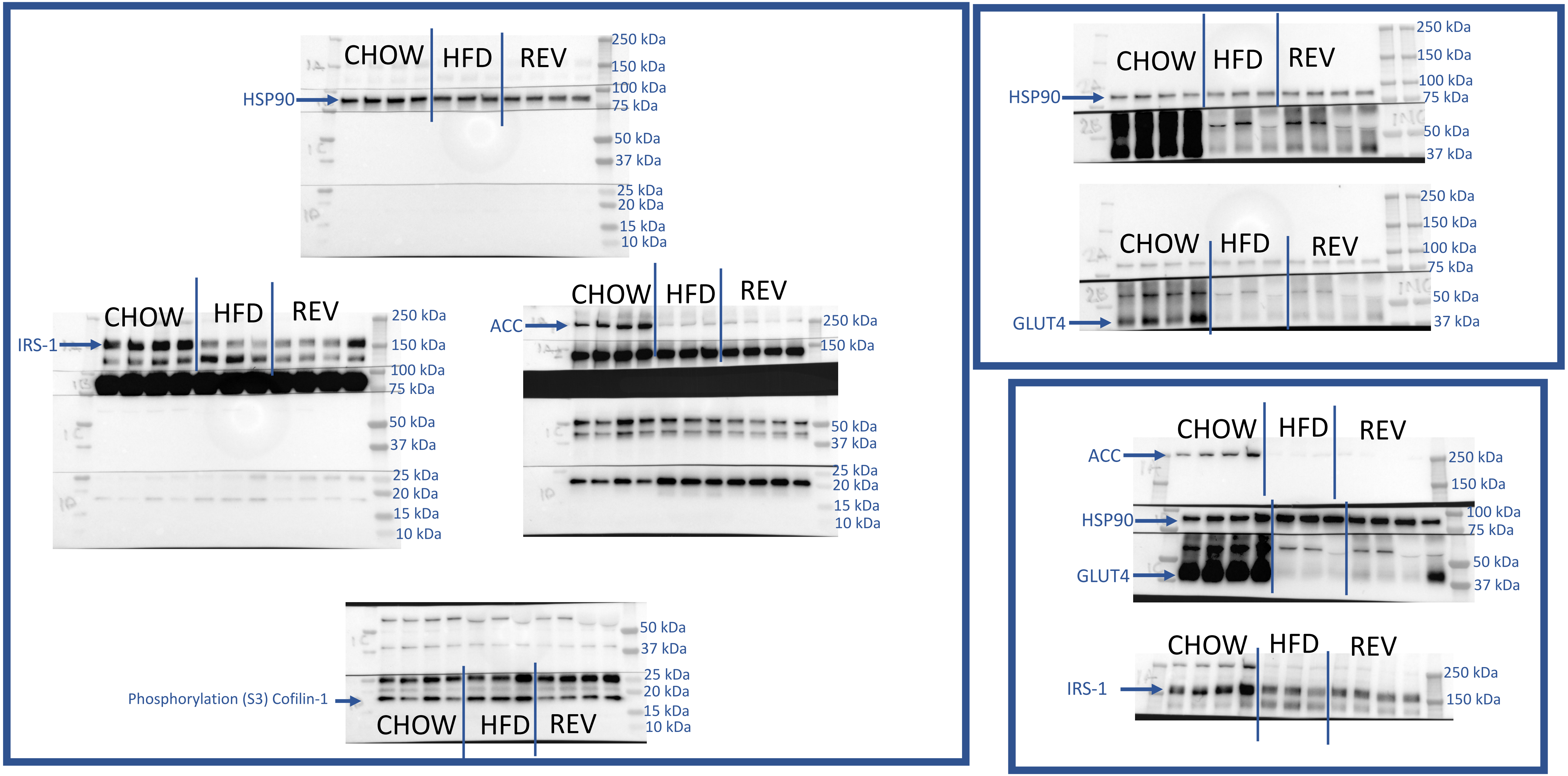

Supplement Fig. 6C

Western Blot uncropped, merged with ladder

Non-stimulated EPI adipocytes 12w group: p-IRS-1, p-AKT, p-AS160

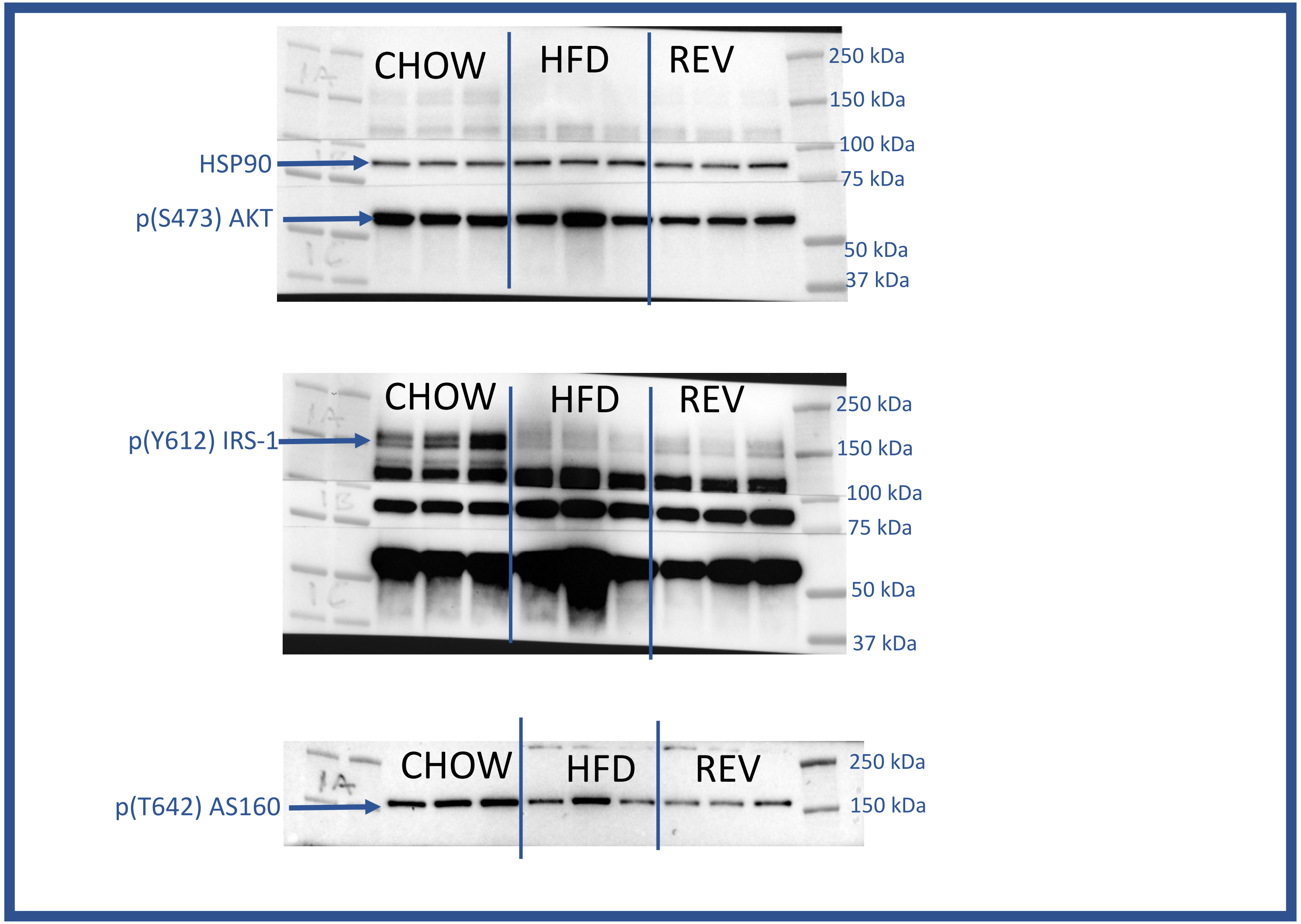

Supplement Fig. 6D

Western Blot uncropped, merged with ladder

Non stimulated ING adipocytes 12w group: p-IRS-1, p-AKT, p-AS160

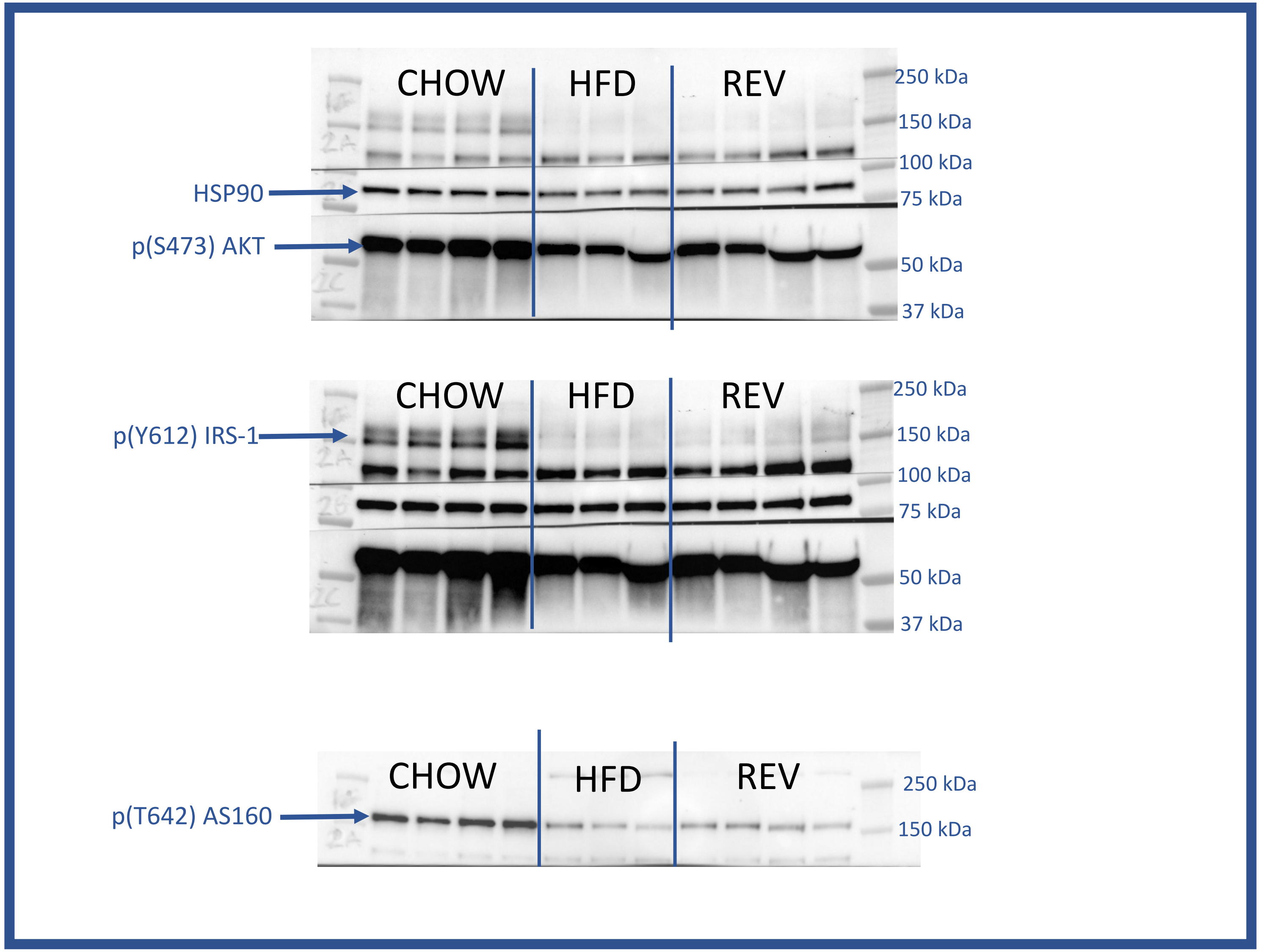

Supplement Fig. 6E

Western Blot uncropped, merged with ladder

EPI adipocytes 4w group: IRS-1, ACC, GLUT4

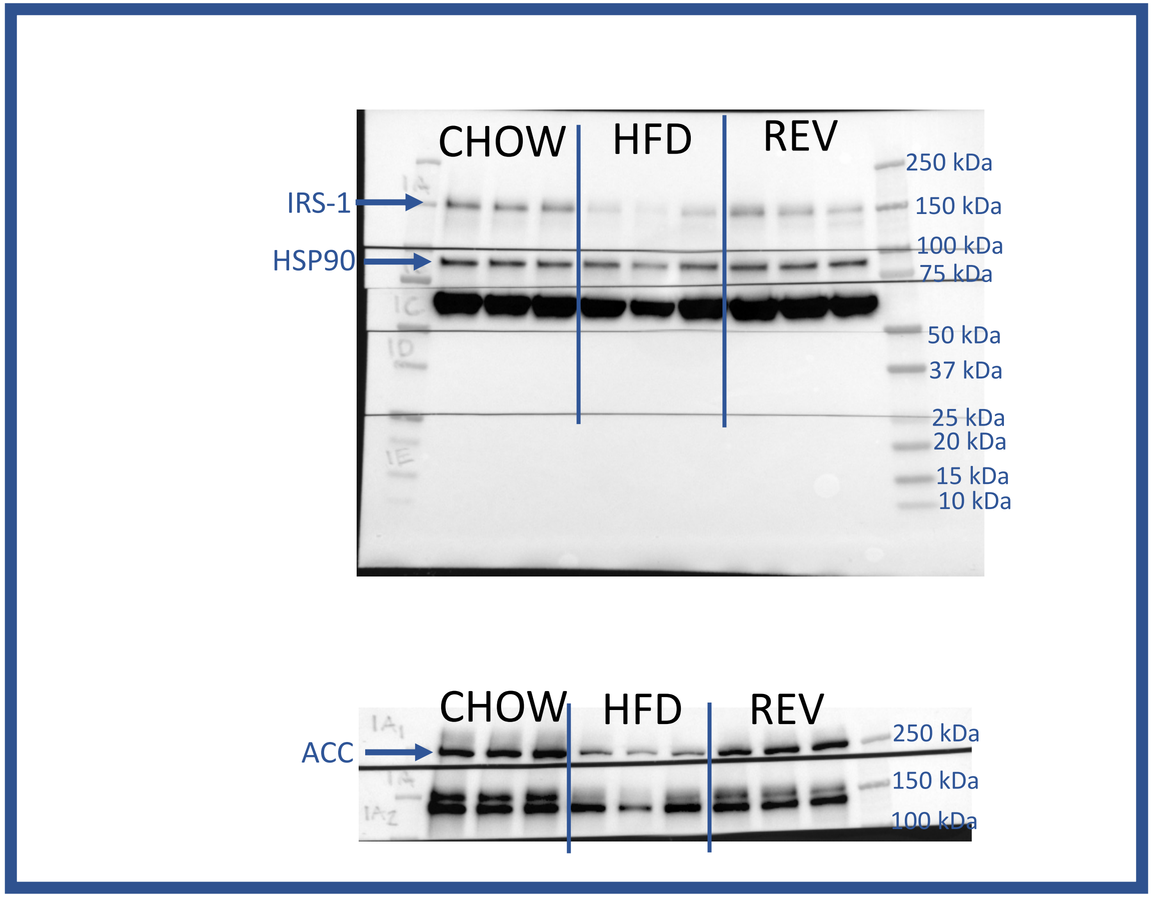

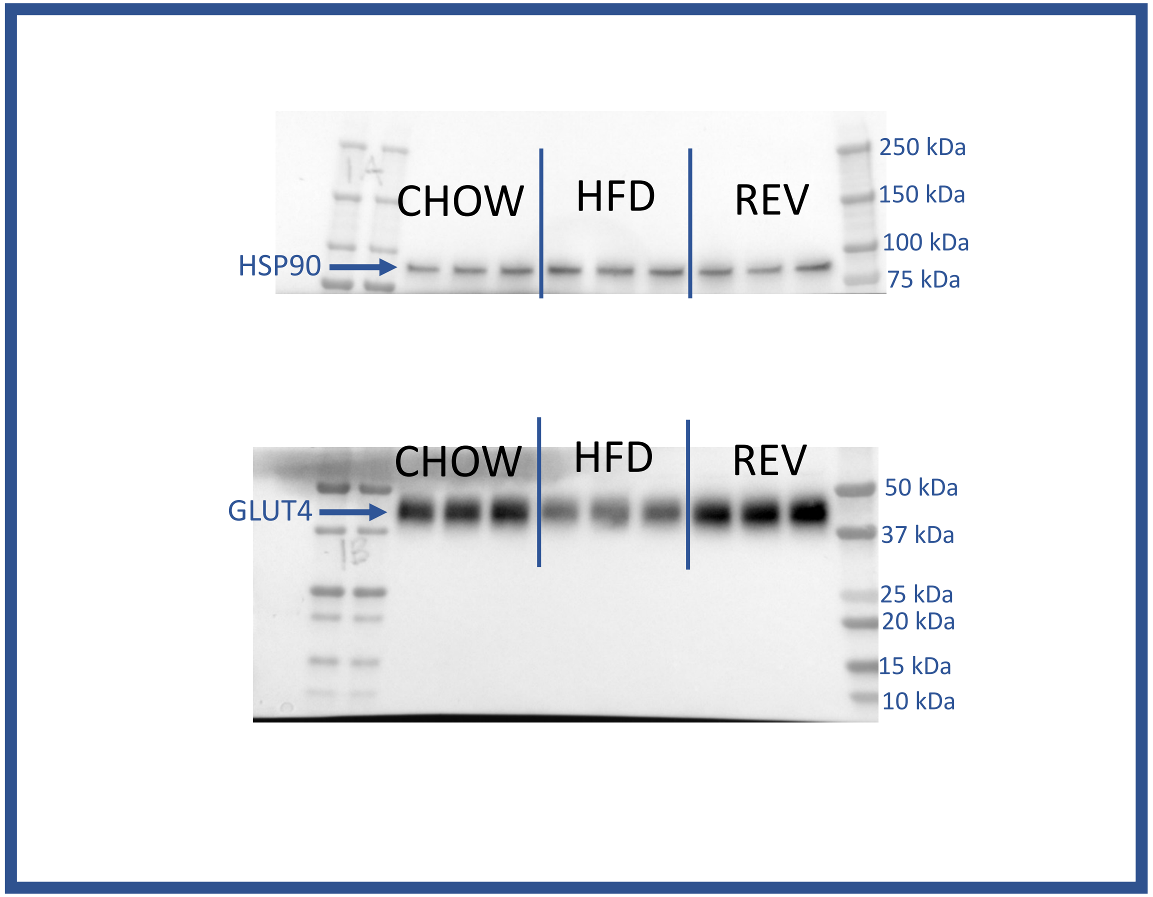

Supplement Fig. 6F

Western Blot uncropped, merged with ladder

EPI 8w group: IRS-1, ACC, GLUT4

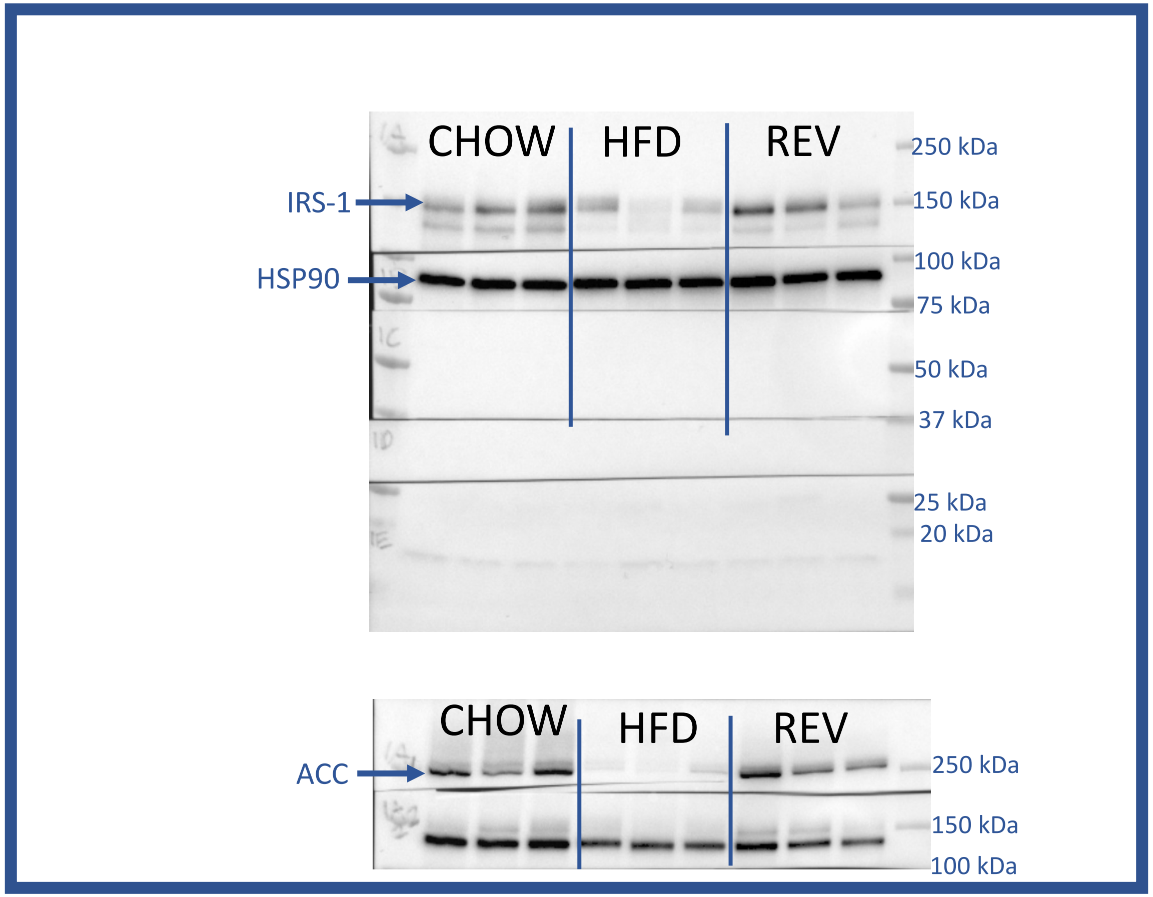

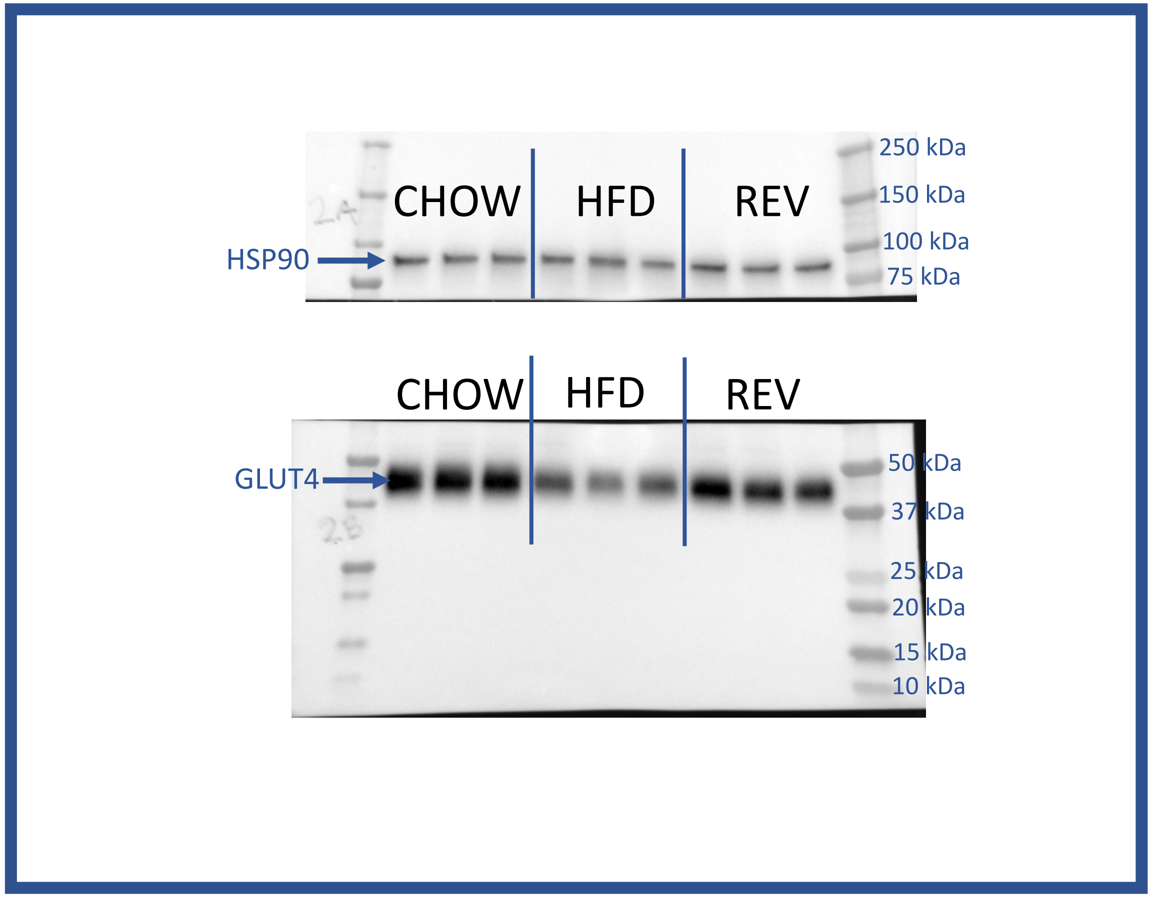

Supplement: Supplementary file 3 [file DataSheet1.PDF]
